# Cardiovascular effects of exercise training in spontaneously hypertensive rats: A systematic review and meta‐analysis

**DOI:** 10.14814/phy2.70794

**Published:** 2026-03-03

**Authors:** Stephen W. Luckey, Rohan Sethi, Natalie S. Crouse, Kayla M. Meredith, Rachael Bush

**Affiliations:** ^1^ Department of Biology Seattle University Seattle Washington USA

**Keywords:** age, exercise, hypertension, intensity, sex, spontaneously hypertensive rats

## Abstract

Exercise is recommended as a nonpharmacological intervention to lower blood pressure in individuals with hypertension, yet optimal exercise parameters and physiological factors influencing its effectiveness remain incompletely understood. We conducted a meta‐analysis to quantify the effects of treadmill‐based exercise training in spontaneously hypertensive rats (SHR), a well‐established animal model of hypertension. A systematic literature search identified 116 studies reporting systolic blood pressure (SBP), mean arterial pressure (MAP), or resting heart rate (RHR). Random‐effects models showed that exercise significantly improved cardiovascular outcomes, with large effect sizes and substantial heterogeneity observed for SBP (−1.19 (Hedges' *g*); 95% CI: −1.43 to −0.94; *p* < 0.001; *I* ^2^ = 81.2%), MAP (−1.06 (Hedges' *g*); 95% CI: −1.27 to −0.85; *p* < 0.001; *I* ^2^ = 66.2%), and RHR (−1.02; 95% CI: −1.23 to −0.81; *p* < 0.001; *I* ^2^ = 71.6%). Subgroup analyses revealed that exercise intensity, sex, and age partially accounted for SBP variability. For MAP, heterogeneity was associated with exercise intensity, sex, and duration of exercise training, while RHR was driven by exercise intensity alone. These findings confirm the benefits of treadmill‐based exercise on cardiovascular health in SHRs and demonstrate the importance of physiological and training‐related factors in modulating the cardiovascular response.

## 1 INTRODUCTION

Hypertension is a major risk factor for cardiovascular disease (CVD) and premature mortality, both in the United States (Cheng et al., 2014; Danaei et al., 2009) and globally (Rapsomaniki et al., 2014). The prevalence of hypertension in the United States is high, with nearly half of all adults aged 18 and over currently living with the condition (Muntner et al., 2020; Ostchega et al., 2020). Moreover, hypertension‐related CVD deaths in the United States increased significantly from 2010 to 2019, particularly among middle‐aged and older adults (Vaughan et al., 2022). Substantial evidence indicates that sex‐based differences in hypertension exist, with men exhibiting higher prevalence at younger ages (20–34 years), while women show greater rates in older age groups (>60 years) (Ryan et al., 2024).

Hypertension is a modifiable disease that can be effectively managed by both nonpharmacological therapies and antihypertensive medications (Whelton et al., 2018). Although extensive evidence has shown that antihypertensive medications reduce the risk for cardiovascular disease and mortality rates (Bundy et al., 2017; Ettehad et al., 2016), only a minority of individuals with hypertension have adequate blood pressure control (Chobufo et al., 2020; Ritchey et al., 2018), and recent data suggest control rates are declining (Muntner et al., 2020). Regular physical activity is widely recognized as an effective non‐pharmacological strategy for managing hypertension and lowering blood pressure, with decades of research consistently reporting that increased physical activity significantly lowers blood pressure in most individuals with hypertension (Cornelissen & Smart, 2013; Hagberg et al., 2000; Pescatello et al., 2004; Tsai et al., 2004; Whelton et al., 2002, 2018). As a result, both U.S. and global clinical guidelines recommend a minimum of 150 min of moderate intensity aerobic exercise or 75 min of vigorous aerobic exercise per week for the prevention and management of hypertension (Unger et al., 2020; Whelton et al., 2002). Despite its overall efficacy, there is a growing body of evidence indicating that exercise has varying effects on lowering blood pressure in some individuals with hypertension (Ferreira et al., 2024; Pescatello & Kulikowich, 2001; Xi et al., 2024). For example, some individuals experience no reduction in blood pressure following dynamic, moderate‐intensity exercise training programs typically lasting 18 weeks, as reported in prior meta‐analysis (Pescatello & Kulikowich, 2001), and, paradoxically, some individuals with prehypertension experienced an increase in blood pressure following 5 months of aerobic, resistance, or combined aerobic and resistance exercise training (Moker et al., 2014). Therefore, current research efforts are focused on understanding how specific characteristics of the exercise training program (e.g., type, intensity, and duration), as well as physiological characteristics including age and sex influence the effectiveness of exercise as a therapeutic intervention for hypertension.

To better understand the pathological features and progression of hypertension, experimental animal models of hypertension have been utilized. Among these, the spontaneously hypertensive rat (SHR) model is a well‐established animal model of hypertension, due to its close resemblance to the progression of human hypertension in terms of disease development and pathology (Doggrell & Brown, 1998). In SHRs, blood pressure begins to rise progressively starting around 5 weeks of age, with fully established hypertension typically observed by 15 weeks (Hom et al., 2007; Okamoto & Aoki, 1963). In addition to elevated blood pressure, this model of hypertension reliably reproduces other key pathological features observed in human hypertension including left ventricular hypertrophy and fibrosis, increased inflammation and apoptosis, and contractile dysfunction, as reviewed by Teles et al. (2023). Finally, similar to human hypertension, the SHR model exhibits sex‐specific differences in the development and progression of hypertension (as reviewed in Elmarakby & Sullivan, 2021).

The SHR model has also been extensively employed to investigate the effects of exercise training on the development and progression of hypertension. Overall, findings from this body of research indicate that exercise exerts beneficial effects on key cardiovascular parameters including systolic blood pressure (SBP), mean arterial pressure (MAP), and resting heart rate (RHR) as well as on the underlying pathophysiological mechanisms involved in the onset and maintenance of hypertension (Teles et al., 2023). However, similar to human studies, the physiological responses to exercise in SHRs exhibit variation. To better understand this variability, recent investigations in SHRs have begun to explore how different exercise modalities and physiological characteristics influence the effectiveness of exercise interventions. For example, a meta‐analysis by Schluter et al. (2010) reported that exercise reduced SBP in SHRs only prior to established hypertension and primarily after shorter durations of training. In contrast, these researchers determined that RHR was reduced regardless of age, sex, or exercise duration. Regarding exercise intensity, studies that directly compared low‐ or moderate‐intensity to high‐intensity exercise training in SHRs consistently found that high‐intensity exercise was less effective in improving cardiovascular outcomes (Chen et al., 2015; Tipton et al., 1983; Veras‐Silva et al., 1997), and in some cases, was associated with exacerbation of cardiovascular disease (Luo et al., 2021; Ye et al., 2019). Furthermore, studies involving female SHRs often reported attenuated cardiovascular adaptations in response to exercise (Amaral et al., 2008; Coimbra et al., 2008; Edwards & Diana, 1978; Kolwicz et al., 2007; Libonati et al., 2011; MacDonnell et al., 2005; Reger et al., 2006; Renna et al., 2006, 2007). Collectively, these findings highlight that, much like in human hypertension, the variability in exercise responses within the SHR model remains inadequately understood and emphasizes the need for further research.

Despite the extensive use of the SHR model in preclinical hypertension research, no prior meta‐analysis has systematically quantified the effects of treadmill‐based training on cardiovascular outcomes in this model. In addition, key sources of heterogeneity have not been thoroughly examined, limiting our understanding of how these factors influence exercise responsiveness. To address this gap, we conducted a systematic review and meta‐analysis to evaluate the effects of treadmill training on SBP, MAP, and RHR in SHRs. We also examined how both training‐related variables (exercise intensity and duration) and biological variables (sex and age) moderate these outcomes, with the goal of informing the design of more effective exercise interventions tailored to different hypertensive populations. Ultimately, the findings from this study not only support evidence‐based exercise prescriptions but also highlight critical gaps in the literature that should be addressed in future research.

## 2 MATERIALS AND METHODS

### 2.1 Data sources and search strategy

The present meta‐analysis was conducted in accordance with the Preferred Reporting Items for Systematic Reviews and Meta‐Analyses (PRISMA) guidelines (Page et al., 2021) (See Figure S1 for the PRISMA checklist). A systematic literature search was performed by two reviewers (K.M. and S.L.), including articles published between 1995 and August 2021. Relevant publications were identified through searches in SciFinder (which includes Pubmed/MEDLINE) (*n* = 538), CINAHL (*n* = 19), SPORTDiscus (*n* = 36), EMBASE (*n* = 255) using the keywords “spontaneously hypertensive rats” and “exercise.” One additional eligible study was identified by reviewing references of retrieved articles. Only English‐language, peer‐reviewed publications were included.

### 2.2 Study selection

**FIGURE 1:**
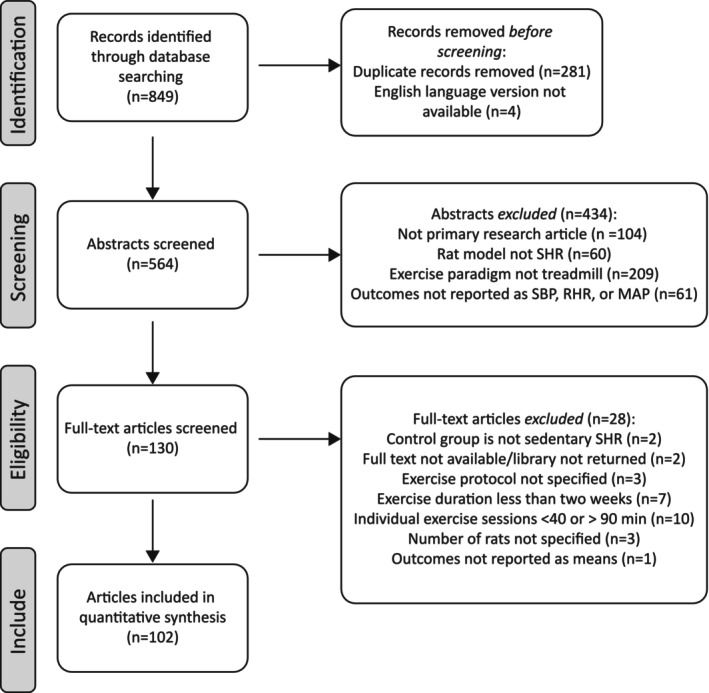
Flow chart of the literature search and study selection process including the different phases of the systematic review.

A total of 849 articles were identified through the search process. Abstracts were independently screened by three reviewers (K.M., N.C., and S.L.). During the abstract review, articles were included if they were primary research articles utilizing the SHR model, employed treadmill exercise protocols with a training period of ≥2 weeks and individual exercise sessions lasting 40–90 min, and reported outcomes for SBP, MAP, and RHR. A total of 130 articles underwent full‐text review, with each independently evaluated by the same three reviewers (K.M., N.C., and S.L.). Articles were excluded based on predefined criteria (Figure 1), and all disagreements were resolved collaboratively.

### 2.3 Data extraction

Data extraction was performed independently by three reviewers (K.M., N.C., and S.L.). Extracted data included authors' names, publication year, sample sizes for exercise and sedentary groups, sex of the rats, age at the onset of exercise training (weeks), training duration (weeks), training intensity expressed as either treadmill speed (m·min^−1^) or percentage of maximal oxygen consumption (%V̇O_2_max), type of instrumentation used to measure cardiovascular outcomes, and the mean and standard deviation (SD) or standard error (SE) for each variable. SE values were converted to SD for analyses. For progressive training protocols, the final maximal workload and training speed were used. When data were presented only graphically, the mean and variability were extracted using ImageJ (ImageJ, https://imagej.net/ij/index.html) by two independent reviewers (N.C. and R.S.), and averaged for analyses. Some publications included multiple experimental groups (e.g., different exercise intensities or separate male and female cohorts) within a single study. In these cases, each experimental group was treated as an independent comparison in the meta‐analysis. In articles with multiple exercise groups but a single sedentary control group, the control group sample size was proportionally divided among exercise groups, following established methods (Vesterinen et al., 2014). All cardiovascular outcome measures (SBP, MAP, RHR) were analyzed as post‐training values. Risk of bias for individual studies was assessed using the SYRCLE Risk of Bias tool for animal studies (Hooijmans et al., 2014). The SYRCLE tool evaluates 10 domains across five categories of bias. Each domain was rated as low risk, high risk, and unclear risk based on information reported in the published articles.

### 2.4 Statistical analysis

All meta‐analyses were performed using Comprehensive Meta‐Analysis Version 4 (Biostat, Inc., Englewood, NJ, USA), and summary figures were generated with Prism 10 (GraphPad Software, La Jolla, CA, USA). The standardized mean difference (Hedges' *g*) and corresponding 95% confidence interval (CI) were calculated for each study to quantify the effect of exercise training versus sedentary controls. Negative effect size values indicate a beneficial effect of exercise. Effect sizes were interpreted as small (0.20), medium (0.50), and large (0.80) according to Cohen's criteria (Cohen, 1992). A random‐effects model was used to pool data and estimate the overall effect size. Forest plots were generated to visually represent study‐level and pooled effects. Sensitivity analyses indicated that no single study disproportionately affected the pooled results. Heterogeneity was assessed using Cochran's *Q* statistic, degrees of freedom, *p* value, the *I* ^2^ statistic, and Tau^2^.

To explore sources of heterogeneity, subgroup analysis and meta‐regression were performed. Subgroups examined sex, age at the start of exercise training, exercise intensity, and training duration. Based on prior literature, exercise intensity was classified using treadmill speed or V̇O_2_max (L·min^−1^), as previously described (Bedford et al., 1979; Gava et al., 1995; Luo et al., 2021; Wang et al., 2020; Ye et al., 2019): low intensity (≤17 m·min^−1^ or <60% V̇O_2_max), moderate intensity (18–25 m·min^−1^ or 60%–75% V̇O_2_max), and high intensity (≥25 m·min^−1^ or ≥75% V̇O_2_max). In cases where protocol details overlapped intensity thresholds, studies were assigned to the most appropriate category based on the predominant characteristics reported. Age was grouped according to the stage of hypertension in SHRs: prehypertensive (≤6 weeks), developing hypertension (7–14 weeks), and hypertensive (≥15 weeks) (Hom et al., 2007; Okamoto & Aoki, 1963). Training duration was categorized as <8 weeks, 8–12 weeks, and ≥13 weeks. Meta‐regression using a random‐effects model quantified the proportion of between‐study variance explained by each moderator (*R* ^2^ analog).

Publication bias was assessed using funnel plots with Egger's regression test (Egger et al., 1997). Funnel plots were generated by plotting Hedges' *g* against the standard error for each study. In the presence of asymmetry, missing studies were imputed using the Duval and Tweedie trim and fill method to estimate an adjusted effect size. Studies lacking moderator data were excluded from subgroup and meta‐regression analyses but retained in the overall pooled analysis when data were available.

## 3 RESULTS

### 3.1 Literature search

A systematic literature search identified studies published between 1995 and August 2021. Figure 1 presents the flow diagram outlining the search process and study exclusions at each stage of the review. The search yielded 849 articles. After removal of 281 duplicates and four non‐English language articles, 564 unique articles remained. Abstract screening excluded 434 articles for one or more of the following reasons: not a primary research article, not using the spontaneously hypertensive rat (SHR) model, employing an exercise modality other than treadmill training, or not reporting SBP, MAP, or RHR outcomes. Full‐text review excluded 28 articles due to: absence of sedentary control group, full text unavailable, unspecified exercise protocol, exercise duration of <2 weeks, individual exercise sessions <40 min or >90 min, unspecified number of rats, or lack of mean outcome values. In total, 102 articles met all eligibility criteria and were included in the final analysis.

### 3.2 Study population and characteristics

Of the 102 eligible articles, several contained more than one experimental study, distinguished by differences in exercise intensity (Battault et al., 2016; Chen et al., 2015; Gava et al., 1995; Luo et al., 2021; Tipton et al., 1983; Veras‐Silva et al., 1997; Ye et al., 2019; Zhang et al., 2017), sex of the rats studied (Edwards & Diana, 1978; Tipton et al., 1983), or measurement instrumentation (Melo et al., 2003). Each distinct experimental design was treated as a separate study, yielding a total of 116 unique studies in the final analysis. Table 1 summarizes study characteristics, encompassing data from 2251 rats. Among the 116 studies, 86 reported SBP, 61 reported MAP, and 73 reported RHR outcomes.

**TABLE 1:** Study population and characteristics of exercised and sedentary SHR groups with cardiovascular outcomes.

Article^b^	Study population (SHR)			Study characteristics				
	Number per group (sed/ex)	Sex male/female	Age at start of exercise program (weeks)	Training intensity		Duration (weeks)	Measuring instrument	Outcomes measured
				Treadmill speed (m/min)	%VO_2_ max (%)			
Agarwal et al. (2009)	10/10	Male	7	18–20		16	TC	MAP^a^, SBP^a^
Agarwal et al. (2011)	10/10	Male	7	18	60	16	TC	MAP^a^, SBP^a^
Amaral et al. (2001)	6/12	Male	10		50–60	13	CA	MAP^a^, RHR
Amaral et al. (2008)	12/12	Female	8.5		50–60	13	CA	MAP
Battault et al. (2016)	4/8	Male	10		80	6	TC	MAP, RHR, SBP
Battault et al. (2016)	4/8	Male	10		55	6	TC	MAP, RHR, SBP
Bertagnolli et al. (2006)	9/9	Male	15	20		10	CA	MAP^a^, RHR SBP^a^
Bertagnolli et al. (2008)	7/7	Male	15	20		10	CA	MAP^a^, RHR SBP^a^
Bertagnolli et al. (2014)	8/9	Male	15	20		10	CA	MAP, RHR, SBP
Birocale et al. (2016)	7/7	Male	12	18		8	TC	SBP^a^
Blanco‐Rivero et al. (2013)	6/6	Male	12	15–20	55–65	12	TC	SBP^a^
Brum et al. (2000)	8/6	Male	8.5	27		13	CA	MAP^a^, RHR^a^, SBP^a^
Cabrera‐Chávez et al. (2020)	6/6	Male	10	16		20	TC	SBP^a^
Caetano et al. (2010)	6/8	Male	8	20	50–70	10	TC	SBP^a^
Cao et al. (2020)	10/10	Male	5	20		8	TC	SBP^a^
Carneiro‐Junior et al. (2013)	8/8	Male	16		50–60	8	TC	RHR, SBP^a^
Chaar et al. (2015)	15/12	Male	12		50–60	12	CA	MAP^a^, RHR^a^
Chen et al. (2015)	9/17	Male	12	26–28	75–85	8	TC	RHR, SBP^a^
Chen et al. (2015)	9/18	Male	12	18–20	55–65	8	TC	RHR^a^, SBP^a^
Chen et al. (2019)	12/12	Male	12	18–20	55–65	12	TC	SBP^a^
Coimbra et al. (2008)	11/11	Female	10		50–60	12	CA	MAP, RHR, SBP
Crisman & Tomanek (1985)	14/17	Male	6		70–90	10	TC	RHR^a^, SBP^a^
da Costa et al. (2020)	10/10	Male	4		50–60	8	CA	MAP^a^, RHR^a^
de Andrade et al. (2015)	15/14	Male	8		55	13	TC	RHR^a^, SBP^a^
Edwards & Diana (1978)	11/6	Male	NR		40–70	10	TC	MAP^a^
Edwards & Diana (1978)	5/5	Female	NR		40–70	10	TC	MAP^a^
Endlich et al. (2011)	7/6	Male	14	24		8	CA	MAP^a^, RHR, SBP^a^
Felix & Michelini (2007)	9/9	Male	8		50–60	12	CA	MAP, RHR^a^
Ferreira et al. (2010)	9/10	Male	16	16		8	TC	SBP
Fragas et al. (2021)	14/14	Male	13		50–60	4	CA	MAP^a^, RHR^a^
Frank et al. (2016)	10/10	Male	4	20–22		8	TC	RHR, SBP
Galdino et al. (2010)	5/5	Male	12	27		4	TC	SBP
Garcia‐Pinto et al. (2011)	8/8	Male	13	16	55	20	TC	SBP^a^
Gava et al. (Gava et al., 1995)	5/13	Male	NR		55	18	CA	RHR^a^
Gava et al. (1995)	6/12	Male	NR		85	18	CA	RHR
Georgieva et al. (2017)	10/10	Male	18	20		4	TC	RHR
Gomes et al. (Gomes et al., 2019)	8/8	Male	16		50–60	12	TC	MAP^a^, RHR, SBP^a^
Graham & Rush (2004)	12/12	Male	11	21	60–70	6	CA	SBP
Gu et al. (2013)	10/10	Male	10	20		12	NR	RHR, SBP
Gu et al. (Gu et al., 2014)	10/10	Male	10	20		12	NR	RHR, SBP^a^
Guo et al. (2008)	10/9	Male	6	20		12	TC	RHR^a^, SBP^a^
Higa‐Taniguchi et al. (2007)	10/10	Male	8		50–60	13	CA	MAP, RHR^a^
Higa‐Taniguchi et al. (2009)	17/16	Male	8		50–60	13	CA	MAP, RHR^a^
Horta et al. (2005)	5/5	Male	6	16	55	20	TC	SBP^a^
Hou (2020)	12/12	NR	4	30		8	TC	MAP^a^, RHR^a^
Huang et al. (2012)	8/8	NR	NR	27		12	TC	MAP^a^, RHR, SBP^a^
Huang et al. (2018)	8/8	Male	13	27		12	TC	MAP, SBP^a^
Ito et al. (2013)	6/6	Male	5	20	50–60	8	TC	SBP^a^
Jia et al. (2014)	7/7	Male	7	18	60	16	TC	MAP^a^
Jodas et al. (2014)	10/10	Male	13	18.3	70–80	16	TC	SBP
Jordao et al. (2011)	12/12	Male	8		50–60	13	CA	MAP^a^, RHR^a^
Kolwicz et al. (2007)	7/8	Female	16	22		12	TC	RHR, SBP
Kramer et al. (2001)	8/8	Male	5	25		10	NR	MAP^a^, RHR^a^, SBP^a^
Lee et al. (2006)	15/15	Male	6	20		13	TC	RHR^a^, SBP^a^
Lehnen et al. (2011)	8/8	Male	24		50–70	10	TC	SBP^a^
Li et al. (2014)	7/7	Male	12	20		8	TC	SBP^a^
Li et al. (2019)	6/6	Male	12	20	60	8	TC	MAP^a^, RHR^a^, SBP^a^
Liao et al. (2018)	20/20	Male	13	28	55–65	8	TC	MAP^a^, RHR^a^, SBP^a^
Libonati et al. (2011)	19/19	Female	16	25		12	TC	SBP, RHR^a^
Lin et al. (2015)	8/8	Male	8	24		8	TC	MAP^a^, RHR, SBP^a^
Little et al. (2001)	9/9	Male	5	24		11	TC	SBP^a^
Luo et al. (2021)	4/12	Male	8	14	35	14	TC	MAP^a^, RHR^a^, SBP^a^
Luo et al. (2021)	4/12	Male	8	20	50	14	TC	MAP^a^, RHR^a^, SBP^a^
Luo et al. (2021)	4/12	Male	8	26	65	14	TC	MAP^a^, RHR, SBP
MacDonnell et al. (2005)	10/12	Female	17	20–25		12	TC	MAP, RHR^a^, SBP
Martins et al. (2005)	5/5	Male	8		50–60	13	TC	SBP^a^
Masson et al. (2014)	12/13	Male	12	10	50–60	8	CA	MAP^a^, RHR^a^
Masson et al. (2015)	7/7	NR	12		50–60	2	CA	MAP, RHR^a^
Matsuura et al. (2012)	8/8	Male	12	16		20	TC	SBP^a^
Melo et al. (2003)	7/7	Male	8		50–60	13	TC	MAP^a^, RHR^a^
Melo et al. (2003)	7/7	Male	8		50–61	13	CA	MAP^a^
Mi et al. (2019)	3/3	Male	8	20		16	CA	SBP^a^
Minami et al. (2003)	8/8	Male	5	20		11	TC	MAP^a^, RHR^a^, SBP^a^
Miotto et al. (2021)	12/13	Male	13		60	8	TC	RHR, MAP^a^, SBP^a^
Moraes‐Teixeira Jde et al. (2010)	8/8	Male	13	16	55	20	NR	MAP^a^
Pagan et al. (2021)	18/17	Male	56	17		16	TC	SBP
Peng et al. (2019)	8/8	Male	6	18–20	55	16	TC	SBP^a^
Qiu et al. (2018)	6/6	Male	12	18–20	55–65	8	TC	MAP^a^, RHR^a^, SBP^a^
Quiroga et al. (2020)	10/10	Male	35		70–85	8	NR	RHR, SBP
Raimundo Fernades et al. (2020)	7/9	Male	18	18–24		8	CA	RHR^a^, MAP^a^
Reger et al. (2006)	9/10	Female	16	25		16	TC	MAP, RHR, SBP
Ren et al. (2016)	10/10	Male	8	15–20	50–60	12	TC (SBP, MAP) CA (RHR)	MAP^a^, RHR^a^, SBP^a^
Renna et al. (2006)	12/10	Female	16	20–25	60	24	TC	MAP, RHR, SBP
Renna et al. (2007)	8/7	Female	17	20–25	60	24	TC	RHR, SBP
Rodrigues et al. (2018)	8/8	NR	16	18–22	60	8	TC	SBP^a^
Roman‐Campos et al. (2012)	10/10	Male	16	16		8	TC	SBP
Roque et al. (2013)	6/8	Male	12	15–20	55–65	12	NR	RHR^a^, SBP^a^
Rossoni et al. (2011)	6/6	Male	92		50–60	13	TC	RHR^a^, SBP^a^
Sallinen et al. (1996)	11/11	Male	8	25	40–60	23	TC	RHR^a^, SBP
Shi et al. (2015)	12/12	Male	12	18–20	55–65	8	TC	MAP^a^, RHR^a^, SBP^a^
Silva et al. (1997)	5/9	Male	NR	15–20	50	12	CA	MAP^a^, RHR^a^, SBP^a^
Silva et al. (2015)	16/10	Male	11.5		50–60	12	CA	MAP^a^, RHR^a^
Silva et al. (2017)	8/8	Male	12		50–60	8	CA	MAP^a^, RHR^a^
Stern et al. (2012)	8/8	Male	6		50–60	6	TC	MAP^a^
Tipton et al. (1983)	19/19	Female and male	42		40–60	14	TC	SBP^a^
Tipton et al. (1983)	11/12	Female	4		40–60	20	TC	SBP^a^
Tipton et al. (1983)	11/10	Male	4		40–60	20	TC	SBP^a^
Tipton et al. (1983)	11/11	Female	8		>75%	24	TC	SBP
Tipton et al. (1983)	11/10	Male	8		>75%	24	TC	SBP
Tipton et al. (1991)	38/38	Male	6		40–70	14	TC	MAP, RHR, SBP^a^
de Tomaz Castro et al. (2021)	6/6	Male	16	18		8	TC	SBP
Totou et al. (2015)	9/6	Male	22	24		8	TC (SBP) CA (MAP, HR)	MAP, RHR^a^, SBP^a^
Veras‐Silva et al. (1997)	3/7	Male	4	16–20	55	18	NR	MAP^a^, RHR^a^, SBP^a^
Veras‐Silva et al. (1997)	4/8	Male	4	25–30	85	18	NR	MAP, RHR, SBP
Wang et al. (2019)	6/9	Male	6	15–20		5	CA	RHR, SBP^a^
Xia et al. (2021)	8/8	Male	12	18		12	TC	MAP^a^, SBP^a^
Ye et al. (2019)	4/8	Male	13	26–28	75–85	8	TC	SBP^a^
Ye et al. (2019)	4/8	Male	13	18–20	55–65	8	TC	SBP^a^
Yen et al. (1995)	12/12	Male	4	15	65	12	TC	RHR^a^, SBP^a^
Zha et al. (2013)	5/5	Male	8	15–20	50–60	12	TC	MAP^a^, RHR^a^
Zhang et al. (2017)	6/12	Male	12	20		8	CA	MAP^a^, RHR^a^
Zhang et al. (Zhang et al., 2017)	6/12	Male	12	14	40–49	8	CA	MAP^a^, RHR^a^
Zhang et al. (2019)	12/12	Male	13	20	55–65	8	TC	MAP^a^, RHR^a^, SBP^a^
Zhang et al. (2020)	24/24	Male	12	20	55–65	12	TC	MAP^a^, SBP^a^
Ziada et al. (2005)	12/12	Male	12	20		10	TC	RHR^a^, SBP^a^
Ziada (2009)	7/7	Male	12	20		10	TC	RHR^a^, SBP

Abbreviations: CA, catheter; H, high; L, low; M, moderate; MAP, mean arterial pressure; NR, not reported; RHR, resting heart rate; SBP, systolic blood pressure; TC, tail cuff.

^a^ Parameter statistically significant from sedentary group.

^b^ Studies are listed alphabetically by first author.

Exercise training protocols reported either the maximal treadmill speed achieved at the end of training, the percentage of maximal exercise capacity, or both. Based on these parameters, each study was classified as low, moderate, or high intensity, as defined in the Methods section. Most studies used moderate‐intensity exercise protocols (*n* = 55), followed by low‐intensity (*n* = 44) and high‐intensity protocols (*n* = 17). Male SHRs were used in most studies (86.2%), whereas only 11 studies (9.5%) included females. Four studies did not report the sex of the rats, and one study included both sexes. The average age at the start of training was 12.2 ± 10.4 weeks (mean ± SD; range 4–92 weeks), indicating that most studies were conducted in younger animals, likely in the earlier stages of disease progression. Training duration, defined as the overall length of the intervention period (e.g., number of weeks), ranged from 2 to 24 weeks (mean 11.8 ± 4.6 weeks). At training onset, female SHRs were approximately the same age as males (12.9 ± 4.5 weeks vs. 11.9 ± 10.5 weeks, mean ± SD). However, female SHRs underwent significantly longer training durations compared with males (16.3 ± 5.4 weeks vs. 11.5 ± 4.2 weeks, *p* < 0.001). Cardiovascular outcomes were primarily measured using noninvasive tail‐cuff plethysmography (66.4%), followed by cardiac catheterization (25.0%). Two studies used both techniques, and eight studies did not specify the measurement technique.

### 3.3 Risk of bias assessment

Risk of bias assessment using the SYRCLE tool revealed variable and often incomplete methodological reporting across the included studies (Figure S2). For selection bias, just over half of studies reported adequate random sequence generation, and 43% reported baseline characteristics. However, allocation concealment was unclear in 100% of the studies. Regarding performance bias, random housing and blinding of investigators were unclear in most studies. For detection bias, 51% of the studies reported random outcome assessment, but blinding of outcome assessors was unclear in all studies (100%). Incomplete outcome data were adequately addressed in less than half of studies, while selective outcome reporting was rated as low risk of bias in all studies (100%). Other potential sources of bias were unclear across all studies.

### 3.4 Systolic blood pressure

**FIGURE 2:**
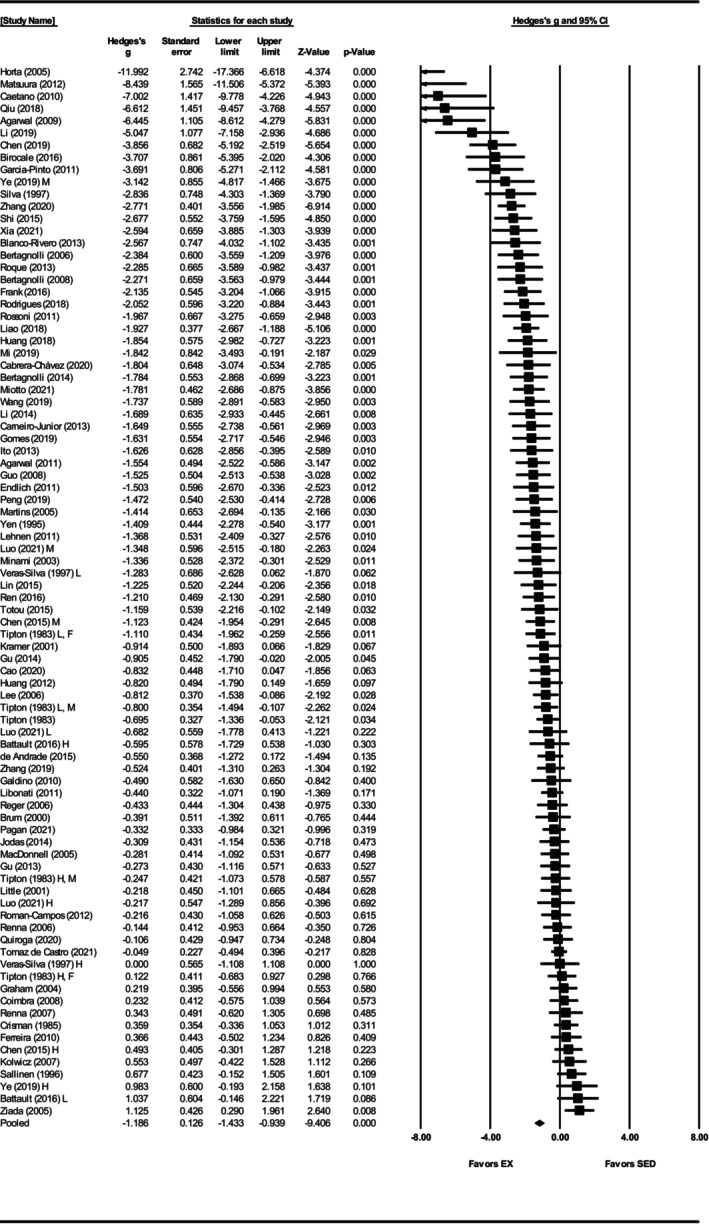
Forest plot of the effects of treadmill exercise training compared with sedentary controls on systolic blood pressure (SBP) in spontaneously hypertensive rats (SHRs). Standardized mean differences (Hedges' *g*) with 95% confidence intervals (CI)s are shown for individual studies. The overall pooled effect size, calculated using a random effects model, is represented by the diamond at the bottom. Values to the left of zero indicate a beneficial effect of exercise training (Favors EX), whereas values to the right indicate a greater response in the sedentary group (Favors SED). Studies with letter designations (L/M/H, low/moderate/high intensity; M/F, male/female) indicate publications with multiple experimental groups, each analyzed separately. *p*‐values displayed as ‘*p* = 0.000’ indicate *p* < 0.001.

Systolic blood pressure was assessed in 86 studies (Agarwal et al., 2009, 2011; Battault et al., 2016; Bertagnolli et al., 2006, 2008, 2014; Birocale et al., 2016; Blanco‐Rivero et al., 2013; Brum et al., 2000; Cabrera‐Chávez et al., 2020; Caetano et al., 2010; Cao et al., 2020; Carneiro‐Junior et al., 2013; Chen et al., 2015, 2019; Coimbra et al., 2008; Crisman & Tomanek, 1985; de Andrade et al., 2015; de Tomaz Castro et al., 2021; Endlich et al., 2011; Ferreira et al., 2010; Frank et al., 2016; Galdino et al., 2010; Garcia‐Pinto et al., 2011; Gomes et al., 2019; Graham & Rush, 2004; Gu et al., 2013, 2014; Guo et al., 2008; Horta et al., 2005; Huang et al., 2012, 2018; Ito et al., 2013; Jodas et al., 2014; Kolwicz et al., 2007; Kramer et al., 2001; Lee et al., 2006; Lehnen et al., 2011; Li et al., 2014, 2019; Liao et al., 2018; Libonati et al., 2011; Lin et al., 2015; Little et al., 2001; Luo et al., 2021; MacDonnell et al., 2005; Martins et al., 2005; Matsuura et al., 2012; Mi et al., 2019; Minami et al., 2003; Miotto et al., 2021; Pagan et al., 2021; Peng et al., 2019; Qiu et al., 2018; Quiroga et al., 2020; Reger et al., 2006; Ren et al., 2016; Renna et al., 2006, 2007; Rodrigues et al., 2018; Roman‐Campos et al., 2012; Roque et al., 2013; Rossoni et al., 2011; Sallinen et al., 1996; Shi et al., 2015; Silva et al., 1997; Tipton et al., 1983, 1991; Totou et al., 2015; Veras‐Silva et al., 1997; Wang et al., 2019; Xia et al., 2021; Ye et al., 2019; Yen et al., 1995; Zhang et al., 2019, 2020; Ziada, 2009, 2005). Exercise training significantly reduced SBP in SHRs, with a large pooled effect size (Hedges' *g* = −1.19, 95% CI: −1.43 to −0.95, *p* < 0.001) (Figures 2 and 12a). Significant heterogeneity was detected (*Q*‐value = 450.93, *τ* ^2^ = 1.04, and *I* ^2^ = 81.15%), indicating substantial variability among studies (Figure 12a). Sensitivity analysis confirmed that no individual disproportionately influenced the pooled effect. Visual inspection of the funnel plot revealed asymmetry, with a disproportionate number of studies to the left of the mean and no studies to the right (Figure S3). Egger's test supported this bias (intercept = −5.20, *p* < 0.001). Using the trim‐and‐fill method, 25 studies were imputed, adjusting the effect size to −0.50 (95% CI: −0.60 to 0.40).

**FIGURE 3:**
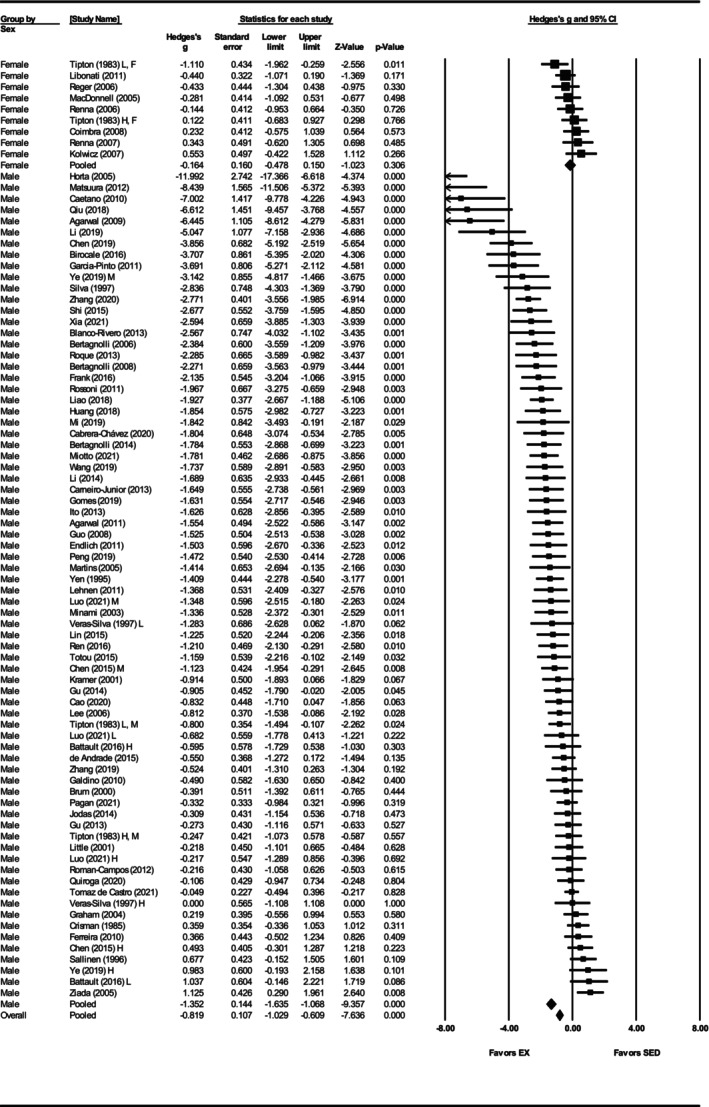
Forest plot depicting the effect of sex on systolic blood pressure (SBP) in spontaneously hypertensive rats (SHRs), with subgroup analysis evaluating differences between males and females. Standardized mean differences (Hedges' *g*) with 95% confidence intervals (CI)s are shown for individual studies. The overall pooled effect size, calculated using a random effects model, is represented by the diamond at the bottom. Values to the left of zero indicate a beneficial effect of exercise training (Favors EX), whereas values to the right indicate a greater response in the sedentary group (Favors SED). Studies with letter designations (L/M/H, low/moderate/high intensity; M/F, male/female) indicate publications with multiple experimental groups, each analyzed separately. *p*‐values displayed as ‘*p* = 0.000’ indicate *p* < 0.001.

**FIGURE 4:**
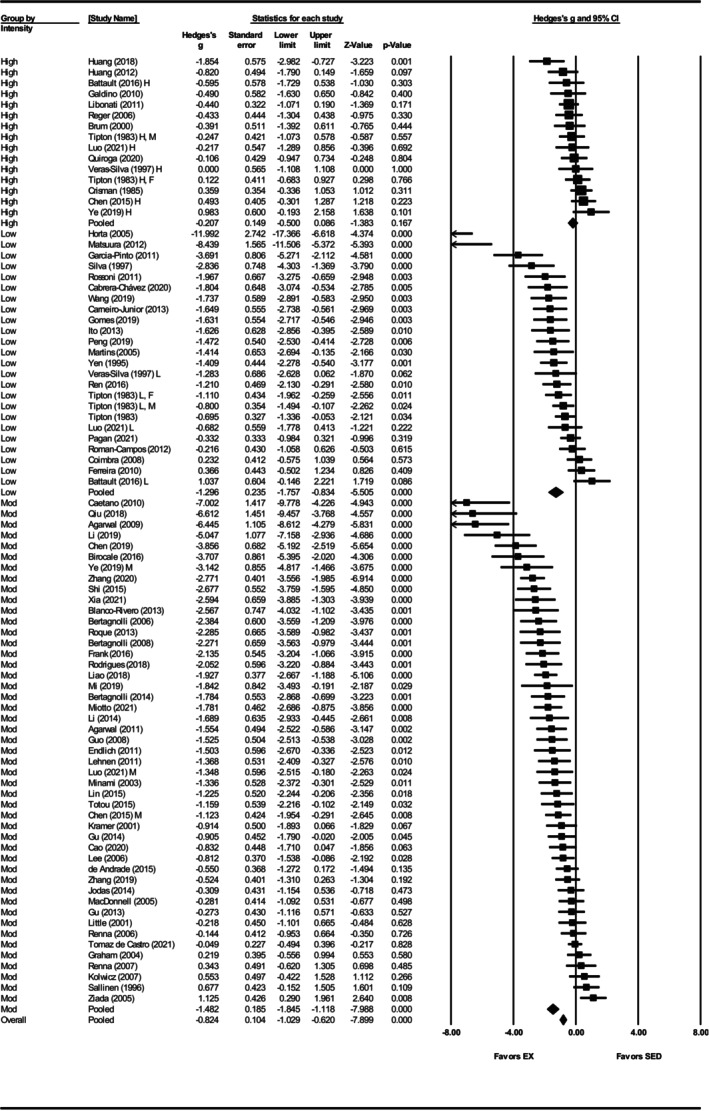
Forest plot depicting the effect of exercise intensity on systolic blood pressure (SBP) in spontaneously hypertensive rats (SHRs), with subgroup analysis evaluating the impact of low‐, moderate‐, and high‐intensity exercise. Standardized mean differences (Hedges' *g*) with 95% confidence intervals (CI)s are shown for individual studies. The overall pooled effect size, calculated using a random effects model, is represented by the diamond at the bottom. Values to the left of zero indicate a beneficial effect of exercise training (Favors EX), whereas values to the right indicate a greater response in the sedentary group (Favors SED). Studies with letter designations (L/M/H, low/moderate/high intensity; M/F, male/female) indicate publications with multiple experimental groups, each analyzed separately. *p*‐values displayed as ‘*p* = 0.000’ indicate *p* < 0.001.

**FIGURE 5:**
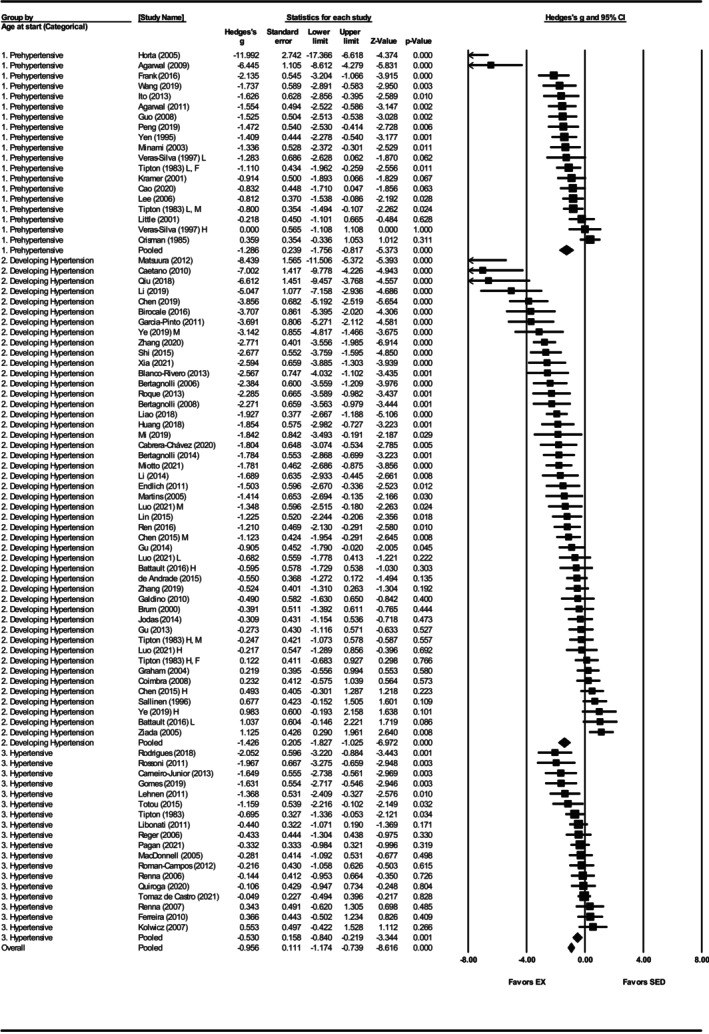
Forest plot depicting the effect of age at the onset of exercise on systolic blood pressure (SBP) in spontaneously hypertensive rats (SHRs), with subgroup analysis evaluating the impact of age at the prehypertensive, developing hypertension, and hypertensive stages. Standardized mean differences (Hedges' *g*) with 95% confidence intervals (CI)s are shown for individual studies. The overall pooled effect size, calculated using a random effects model, is represented by the diamond at the bottom. Values to the left of zero indicate a beneficial effect of exercise training (Favors EX), whereas values to the right indicate a greater response in the sedentary group (Favors SED). Studies with letter designations (L/M/H, low/moderate/high intensity; M/F, male/female) indicate publications with multiple experimental groups, each analyzed separately. *p*‐values displayed as ‘*p* = 0.000’ indicate *p* < 0.001.

To further explore the observed heterogeneity in the SBP data, subgroup analyses were conducted. Sex‐based analysis demonstrated a significant effect of exercise on SBP in male SHRs (Hedges' *g* = −1.35, 95% CI: −1.64 to −1.07, *p* < 0.001), but not in females (Hedges' *g* = −0.16, 95% CI: −0.48 to 0.15, *p* = 0.31) (Figure 3). Notably, only nine studies involved female SHRs, compared with 75 involving males. The difference between sexes was significant (*Q*‐value = 30.31, *p* < 0.001, *I* ^2^ = 81.64%) accounting for 5% of the between‐subgroup variances (*R* ^2^ = 0.05) (Figures 3 and 12a). When grouped by exercise intensity, low (Hedges' *g* = −1.30, 95% CI: −1.76 to −0.83, *p* < 0.001) and moderate intensity groups (Hedges' *g* = −1.48, 95% CI: −1.85 to −1.12, *p* < 0.001) produced greater reductions than high intensity (Hedges' *g* = −0.21, 95% CI: −0.50 to 0.09, *p* = 0.17) (Figures 4 and 12a). Differences between intensities were significant (*Q*‐value = 33.63, *p* < 0.001, *I* ^2^ = 81.15%) (Figure 12a). This relationship accounted for 7% of the differences between subgroups (*R* ^2^ = 0.07). All three age groups showed significant reductions, with greater effects in prehypertensive (Hedges' *g* = −1.29, CI: −1.76 to −0.82, *p* < 0.001) and developing hypertension groups (Hedges' *g* = −1.43, CI: −1.83 to −1.03, *p* < 0.001) than in hypertensive SHRs (Hedges' *g* = −0.53, CI: −0.84 to −0.22, *p* < 0.001) (Figures 5 and 12a). Age differences were significant (*Q*‐value = 14.43, *p* < 0.005, *I* ^2^ = 81.30%) and this described 3% of the differences between subgroups (*R* ^2^ = 0.03) (Figure 12a). Significant reductions occurred with 8–12 weeks (Hedges' *g* = −1.35, 95% CI: −1.68 to −1.02, *p* < 0.001) and ≥13 weeks of training (Hedges' *g* = −1.03, 95% CI: −1.43 to −0.64, *p* < 0.001), but not with ≤8 weeks (Hedges' *g* = −0.29, 95% CI: −1.14 to 0.56, *p* = 0.50) (Figures S4 and 12a). Only five studies were included in the ≤8 weeks group, and differences between durations were not statistically significant (*Q*‐value = 5.59, *p* = 0.06) (Figure 12a).

### 3.5 Mean arterial pressure

**FIGURE 6:**
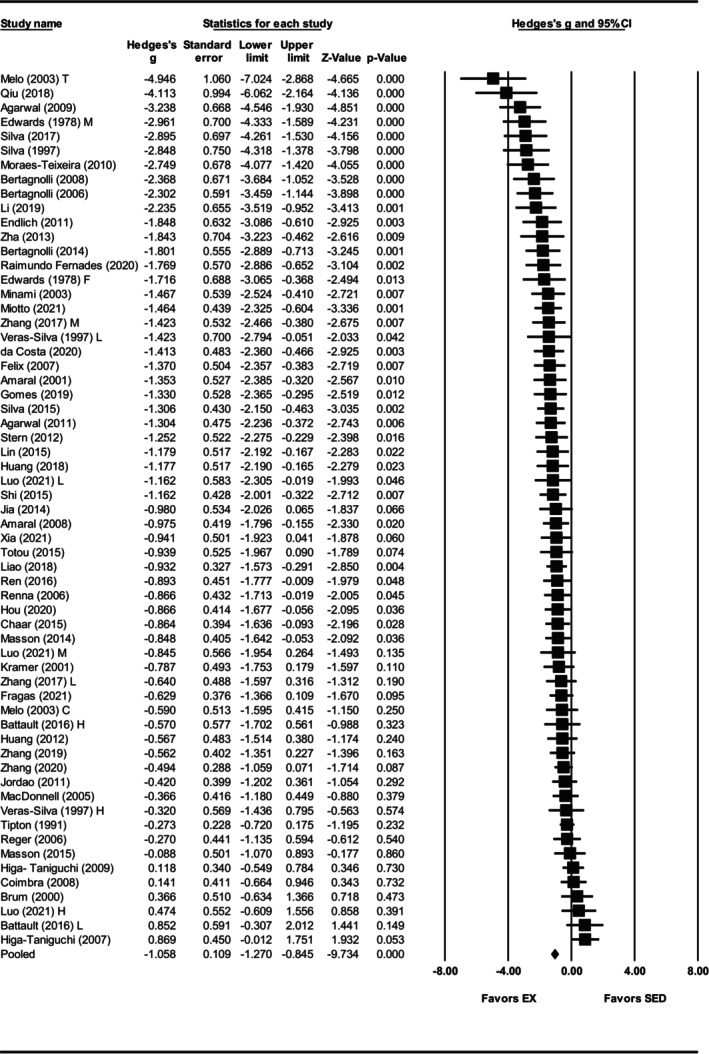
Forest plot of the effects of exercise training compared with sedentary controls on mean arterial pressure (MAP) in spontaneously hypertensive rats (SHRs). Standardized mean differences (Hedges' *g*) with 95% confidence intervals (CI)s are shown for individual studies. The overall pooled effect size, calculated using a random effects model, is represented by the diamond at the bottom. Values to the left of zero indicate a beneficial effect of exercise training (Favors EX), whereas values to the right indicate a greater response in the sedentary group (Favors SED). Studies with letter designations (L/M/H, low/moderate/high intensity; M/F, male/female; C/T, catheter/tail cuff) indicate publications with multiple experimental groups, each analyzed separately. *p*‐values displayed as ‘*p* = 0.000’ indicate *p* < 0.001.

Mean arterial pressure was assessed in 61 studies (Agarwal et al., 2009, 2011; Amaral et al., 2001, 2008; Battault et al., 2016; Bertagnolli et al., 2006; 2008, 2014; Brum et al., 2000; Chaar et al., 2015; Coimbra et al., 2008; da Costa et al., 2020; Edwards & Diana, 1978; Endlich et al., 2011; Felix & Michelini, 2007; Fragas et al., 2021; Gomes et al., 2019; Higa‐Taniguchi et al., 2007, 2009; Hou, 2020; Huang et al., 2012; Huang et al., 2018; Jia et al., 2014; Jordao et al., 2011; Kramer et al., 2001; Li et al., 2019; Liao et al., 2018; Lin et al., 2015; Luo et al., 2021; MacDonnell et al., 2005; Masson et al., 2014, 2015; Melo et al., 2003; Minami et al., 2003; Miotto et al., 2021; Moraes‐Teixeira Jde et al., 2010; Qiu et al., 2018; Raimundo Fernades et al., 2020; Reger et al., 2006; Ren et al., 2016; Renna et al., 2006; Shi et al., 2015; Silva et al., 1997; Silva et al., 2015, 2017; Stern et al., 2012; Tipton et al., 1991; Totou et al., 2015; Veras‐Silva et al., 1997; Xia et al., 2021; Zha et al., 2013; Zhang et al., 2017, 2019, 2020). Exercise training significantly reduced MAP, with a large pooled effect size (Hedges' *g* = −1.06, 95% CI: −1.27 to −0.85, *p* < 0.001) and significant heterogeneity (*Q*‐value = 177.32, *τ* ^2^ = 0.45, *I* ^2^ = 66.16%) (Figures 6 and 12b). Results of the sensitivity analysis showed that removal of any individual study did not substantially change the overall effect estimate. Funnel plots suggested asymmetry, which was supported by Egger's test (intercept = −4.11, *p* < 0.001). Trim and fill analysis added 18 studies, reducing the effect size to −0.63 (95% CI: −0.74 to −0.52) (Figure S5).

**FIGURE 7:**
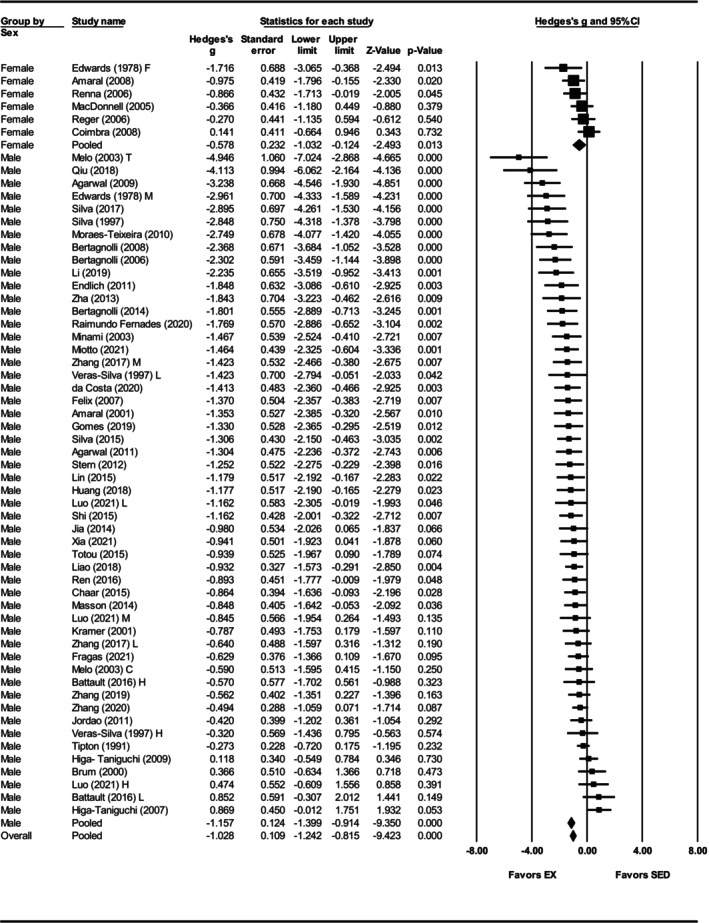
Forest plot depicting the effect of sex on mean arterial pressure (MAP) in spontaneously hypertensive rats (SHRs), with subgroup analysis evaluating differences between males and females. Standardized mean differences (Hedges' *g*) with 95% confidence intervals (CI)s are shown for individual studies. The overall pooled effect size, calculated using a random effects model, is represented by the diamond at the bottom. Values to the left of zero indicate a beneficial effect of exercise training (Favors EX), whereas values to the right indicate a greater response in the sedentary group (Favors SED). Studies with letter designations (L/M/H, low/moderate/high intensity; M/F, male/female; C/T, catheter/tail cuff) indicate publications with multiple experimental groups, each analyzed separately. *p*‐values displayed as ‘*p* = 0.000’ indicate *p* < 0.001.

**FIGURE 8:**
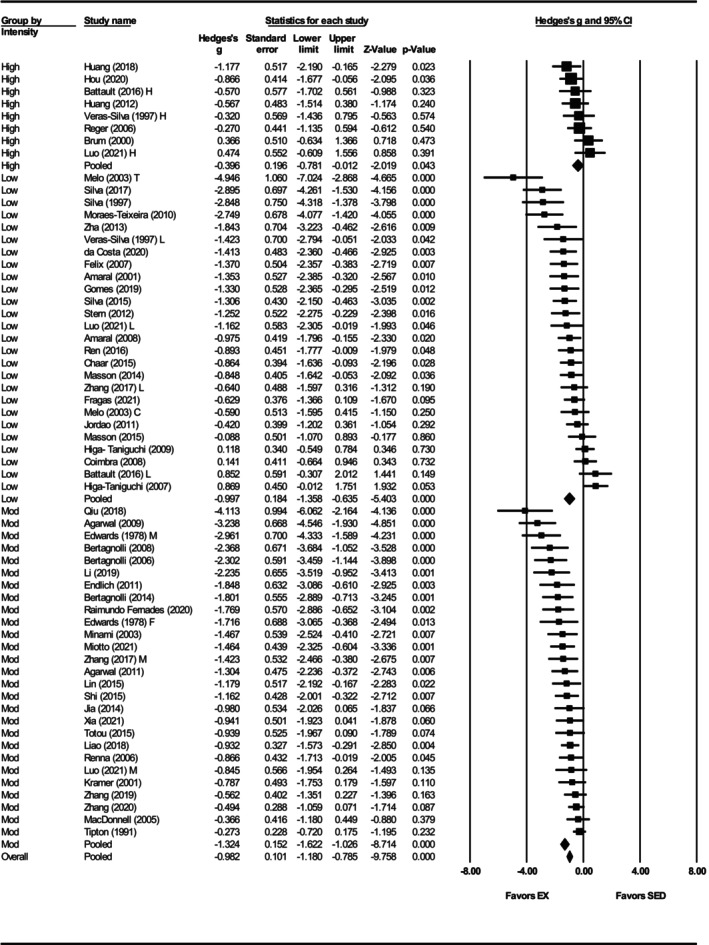
Forest plot depicting the effect of exercise intensity on mean arterial pressure (MAP) in spontaneously hypertensive rats (SHRs), with subgroup analysis evaluating the impact of low‐, moderate‐, and high‐intensity exercise. Standardized mean differences (Hedges' *g*) with 95% confidence intervals (CI)s are shown for individual studies. The overall pooled effect size, calculated using a random effects model, is represented by the diamond at the bottom. Values to the left of zero indicate a beneficial effect of exercise training (Favors EX), whereas values to the right indicate a greater response in the sedentary group (Favors SED). Studies with letter designations (L/M/H, low/moderate/high intensity; M/F, male/female; C/T, catheter/tail cuff) indicate publications with multiple experimental groups, each analyzed separately. *p*‐values displayed as ‘*p* = 0.000’ indicate *p* < 0.001.

**FIGURE 9:**
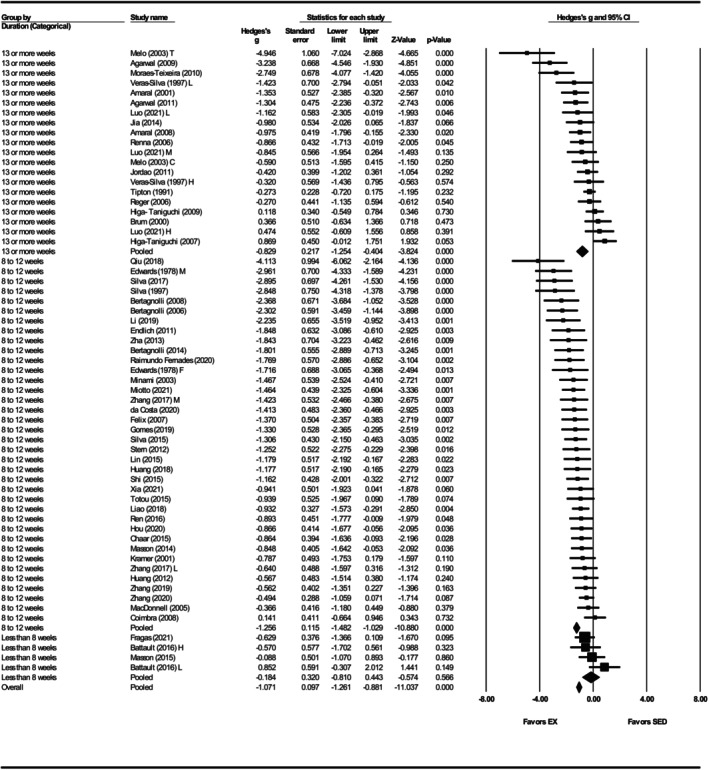
Forest plot depicting the effect of training duration on mean arterial pressure (MAP) in spontaneously hypertensive rats (SHRs), with subgroup analysis evaluating the impact of ≤8 weeks, 9–12 weeks, and ≥13 weeks of training. Standardized mean differences (Hedges' *g*) with 95% confidence intervals (CI)s are shown for individual studies. The overall pooled effect size, calculated using a random effects model, is represented by the diamond at the bottom. Values to the left of zero indicate a beneficial effect of exercise training (Favors EX), whereas values to the right indicate a greater response in the sedentary group (Favors SED). Studies with letter designations (L/M/H, low/moderate/high intensity; M/F, male/female; C/T, catheter/tail cuff) indicate publications with multiple experimental groups, each analyzed separately. *p*‐values displayed as ‘*p* = 0.000’ indicate *p* < 0.001.

Subgroup analysis was performed to explore the sources of heterogeneity in the effect of exercise training on MAP. Both sexes exhibited reductions in MAP with exercise training, but the effect was greater in males (Hedges' *g* = −1.16, 95% CI: −1.40 to −0.91, *p* < 0.001) than in females (Hedges' *g* = −0.58, 95% CI: −1.03 to −0.12, *p* < 0.01), with a significant between sex‐difference (*Q*‐value = 4.86, *p* < 0.05) (Figures 7 and 12b). Strikingly, only six studies involved the female SHRs. For exercise intensity, low (Hedges' *g* = −1.00, 95% CI: −1.36 to −0.64, *p* < 0.001) and moderate (Hedges' *g* = −1.32, 95% CI: −1.62 to −1.03, *p* < 0.001) intensities were more effective than high intensity (Hedges' *g* = −0.40, 95% CI: −0.78 to −0.01, *p* < 0.01) (Figures 8 and 12b). Significant differences were observed between the subgroups (*Q*‐value = 13.98, *p* < 0.005; Figure 12b), with the analysis explaining 5% of the differences (*R* ^2^ = 0.05). Age at the onset of exercise training produced similar reductions across all three groups: prehypertensive (Hedges' *g* = −1.12, 95% CI: −1.56 to −0.67, *p* < 0.001), developing hypertension (Hedges' *g* = −1.01, 95% CI: −1.28 to −0.73, *p* < 0.001), and hypertensive (Hedges' *g* = −0.84, 95% CI: −1.28 to −0.41, *p* < 0.001), with no significant subgroup difference (*Q*‐value = 0.76, *p* = 0.68) (Figures S6 and 12b). It is worth noting that the hypertensive subgroup was limited to six studies. Significant differences were observed when exercise training programs lasted 8–12 weeks (Hedges' *g* = −1.26, 95% CI: −1.48 to −1.03, *p* < 0.001) and ≥13 weeks (Hedges' *g* = −0.83, 95% CI: −1.25 to −0.40, *p* < 0.001), but not with ≤8 weeks (Hedges' *g* = −0.18, 95% CI: −0.81 to 0.44 *p* = 0.57) (Figures 9 and 12b). Notably, only four studies were in the ≤8 weeks group, which may limit interpretability. Training duration differences were significant (*Q*‐value = 11.51, *p* < 0.005) (Figure 12b) and explains 16% of the MAP differences (*R* ^2^ = 0.16).

### 3.6 Resting heart rate

**FIGURE 10:**
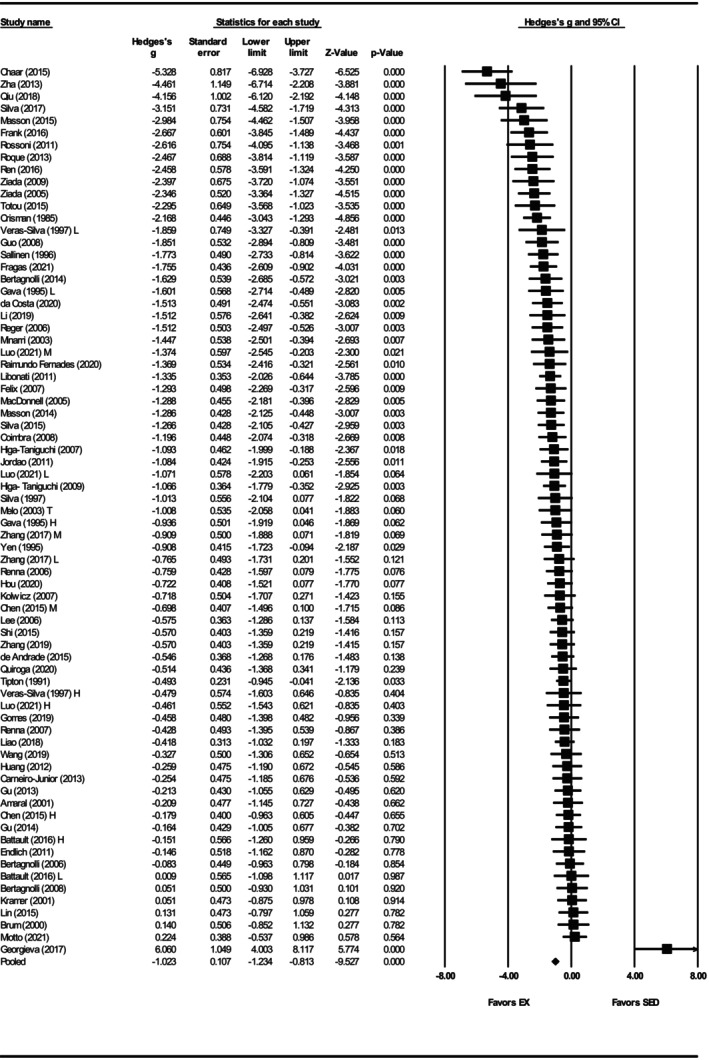
Forest plot of the effects of exercise training compared with sedentary controls on resting heart rate (RHR) in spontaneously hypertensive rats (SHRs). Standardized mean differences (Hedges' *g*) with 95% confidence intervals (CI)s are shown for individual studies. The overall pooled effect size, calculated using a random effects model, is represented by the diamond at the bottom. Values to the left of zero indicate a beneficial effect of exercise training (Favors EX), whereas values to the right indicate a greater response in the sedentary group (Favors SED). Studies with letter designations (L/M/H, low/moderate/high intensity; M/F, male/female; C/T, catheter/tail cuff) indicate publications with multiple experimental groups, each analyzed separately. *p*‐values displayed as ‘*p* = 0.000’ indicate *p* < 0.001.

Resting heart rate was reported in 73 studies (Amaral et al., 2001; Battault et al., 2016; Bertagnolli et al., 2006, 2008, 2014; Brum et al., 2000; Carneiro‐Junior et al., 2013; Chaar et al., 2015; Chen et al., 2015; Coimbra et al., 2008; Crisman & Tomanek, 1985; da Costa et al., 2020; de Andrade et al., 2015; Endlich et al., 2011; Felix & Michelini, 2007; Fragas et al., 2021; Frank et al., 2016; Gava et al., 1995; Georgieva et al., 2017; Gomes et al., 2019; Gu et al., 2013, 2014; Guo et al., 2008; Higa‐Taniguchi et al., 2007, 2009; Hou, 2020; Huang et al., 2012; Jordao et al., 2011; Kolwicz et al., 2007; Kramer et al., 2001; Lee et al., 2006; Li et al., 2019; Liao et al., 2018; Libonati et al., 2011; Lin et al., 2015; Luo et al., 2021; MacDonnell et al., 2005; Masson et al., 2014, 2015; Melo et al., 2003; Minami et al., 2003; Miotto et al., 2021; Qiu et al., 2018; Quiroga et al., 2020; Raimundo Fernades et al., 2020; Reger et al., 2006; Ren et al., 2016; Renna et al., 2006, 2007; Roque et al., 2013; Rossoni et al., 2011; Sallinen et al., 1996; Shi et al., 2015; Silva et al., 1997, 2015, 2017; Tipton et al., 1991; Totou et al., 2015; Veras‐Silva et al., 1997; Wang et al., 2019; Yen et al., 1995; Zha et al., 2013; Zhang et al., 2017, 2019; Ziada, 2009; Ziada et al., 2005). Exercise training significantly reduced RHR (Hedges' *g* = −1.02, 95% CI: −1.23 to −0.81, *p* < 0.001, see Figures 10 and 12c) with large heterogeneity (*Q*‐value = 253.34, *τ* ^2^ = 0.57, *I* ^2^ = 71.58%, see Figure 10). Sensitivity analysis demonstrated that excluding any one study did not disproportionately influence the overall effect size. Funnel plot analysis suggested possible asymmetry, and Egger's test supported the bias (intercept = −2.99, *p* < 0.001) (Figure S7). Trim‐and‐fill analysis estimated the addition of 14 studies, which adjusted the effect size to −0.71 (95% CI: −0.81 to −0.61) (Figure S7).

**FIGURE 11:**
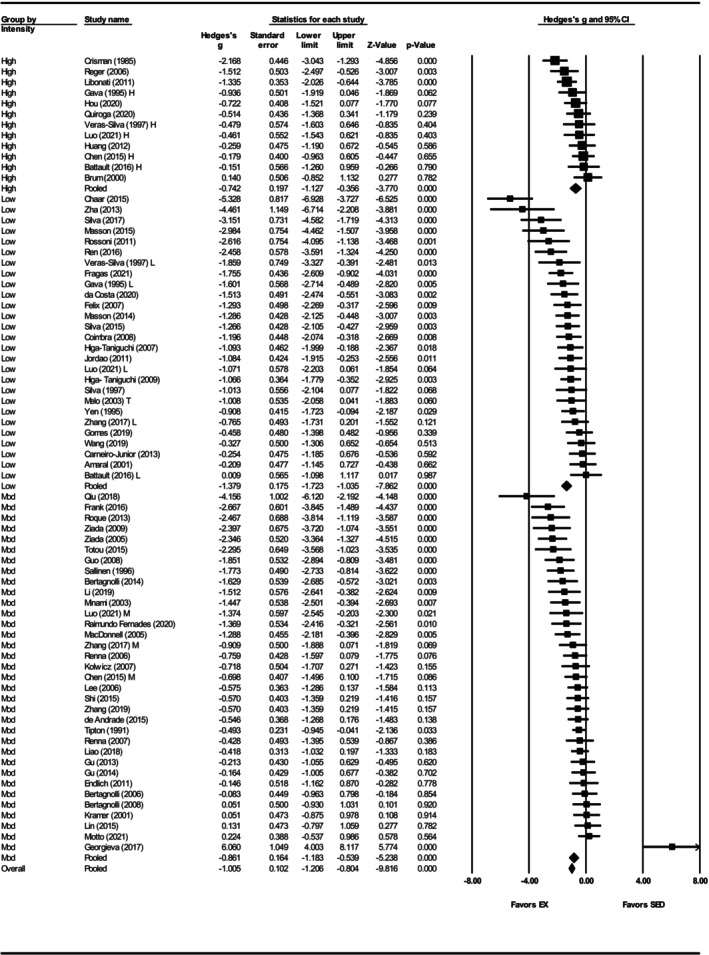
Forest plot depicting the effect of exercise intensity on resting heart rate (RHR) in spontaneously hypertensive rats (SHRs), with subgroup analysis evaluating the impact of low‐, moderate‐, and high‐intensity exercise. Standardized mean differences (Hedges' *g*) with 95% confidence intervals (CI)s are shown for individual studies. The overall pooled effect size, calculated using a random effects model, is represented by the diamond at the bottom. Values to the left of zero indicate a beneficial effect of exercise training (Favors EX), whereas values to the right indicate a greater response in the sedentary group (Favors SED). Studies with letter designations (L/M/H, low/moderate/high intensity; M/F, male/female; C/T, catheter/tail cuff) indicate publications with multiple experimental groups, each analyzed separately. *p*‐values displayed as ‘*p* = 0.000’ indicate *p* < 0.001.

**FIGURE 12:**
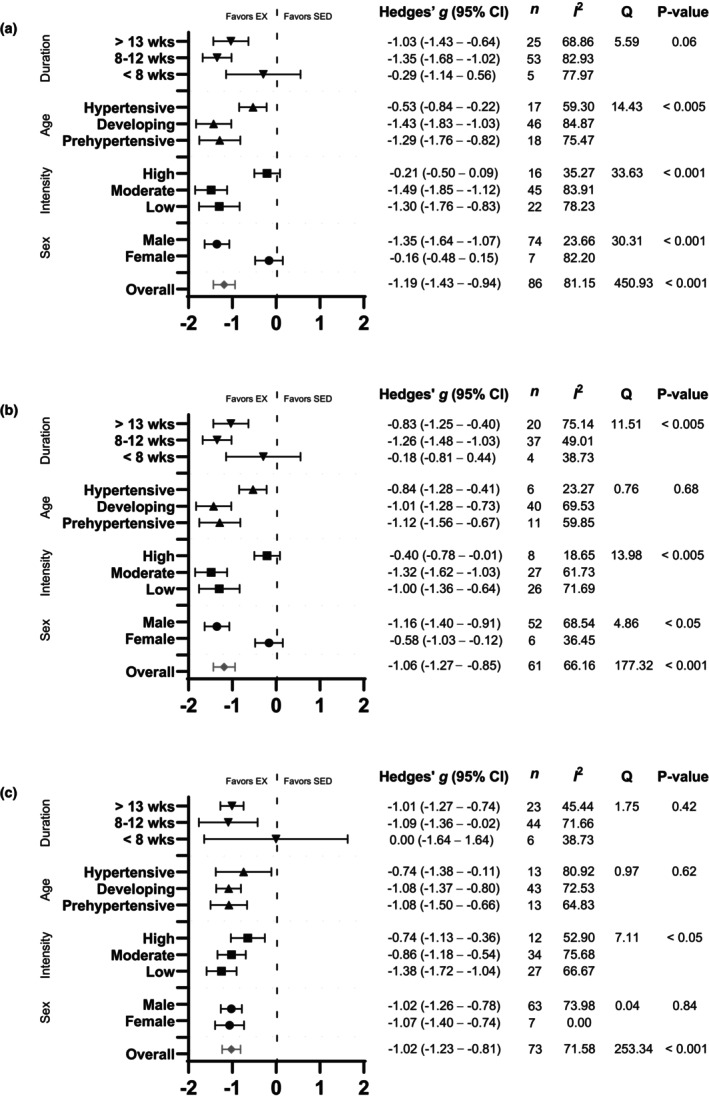
Summary of main meta‐analyses results for (a) systolic blood pressure (SBP), (b) mean arterial pressure (MAP), and (c) resting heart rate (RHR) in spontaneously hypertensive rats (SHRs).

To assess whether training or biological variables influenced RHR outcomes, subgroup analyses were performed. Both sexes reduced RHR (females: Hedges' *g* = −1.07, 95% CI: −1.40 to −0.74, *p* < 0.001; males: Hedges' *g* = −1.02, 95% CI: −1.26 to −0.78, *p* < 0.001) and no significant difference between the groups was observed (*Q*‐value = 0.04 *p* = 0.84) (Figure S8, Figure 12c). Only seven studies included female SHRs, in contrast to 63 studies that used male SHRs. High (Hedges' *g* = −0.74, 95% CI: −1.13 to −0.36, *p* < 0.001) and moderate intensities (Hedges' *g* = −0.86, 95% CI: −1.18 to −0.54, *p* < 0.001) produced smaller effects compared to low intensity (Hedges' *g* = −1.38, 95% CI: −1.72 to −1.04, *p* < 0.001) (Figure 11, Figure 12c), and differences were statistically significant (*Q*‐value = 7.11, *p* < 0.05) (Figure 12c). Exercise intensity explains 5% of the between‐group variance (*R* ^2^ = 0.05). Exercise training reduced RHR across all age groups (prehypertensive: Hedges' *g* = −1.08, 95% CI: −1.50 to −0.66, *p* < 0.001; developing hypertension: Hedges' *g* = −1.08, 95% CI: −1.37 to −0.80, *p* < 0.001; hypertensive Hedges' *g* = −0.74, 95% CI: −1.38 to −0.11, *p* < 0.05), and no significant age‐related differences were observed (*Q*‐value = 0.97, *p* = 0.62) (Figure S9, Figure 12c). Training for ≤8 weeks did not significantly reduce RHR (Hedges' *g* = −0.00, 95% CI: −1.64 to 1.64 *p* = 0.99), though this group only included six studies. In contrast, 8–12 weeks of training (Hedges' *g* = −1.09, 95% CI: −1.36 to −0.02, *p* < 0.001) and ≥13 weeks (Hedges' *g* = −1.01, 95% CI: −1.27 to −0.74, *p* < 0.001) significantly lowered RHR (Figure S10, Figure 12c). However, differences across training durations were not statistically significant (*Q*‐value = 1.75, *p* = 0.42) (Figure 12c).

## 4 DISCUSSION

This study is the first meta‐analysis to systematically evaluate the effects of treadmill‐based exercise training on SBP, MAP, and RHR in SHRs. As hypothesized, our results show that treadmill exercise training produces significant cardiovascular benefits across all three outcomes in this widely used hypertensive model. Male SHRs exhibited greater SBP and MAP reductions than females, although no sex differences were observed for RHR. Low‐ to moderate‐intensity training yielded more substantial cardiovascular improvements than high‐intensity protocols, and longer training durations were associated with greater benefits across all outcomes. Spontaneously hypertensive rats with established hypertension appeared less responsive to exercise. Subgroup analysis revealed that sex, exercise intensity, and age each contribute modest but significant effects on SBP, while sex, exercise intensity, and training duration were important in lowering MAP. Exercise intensity was the only variable significantly associated with RHR changes. This analysis also highlights two key gaps in the literature including the underrepresentation of female and aged SHRs in preclinical exercise research.

### 4.1 Comparison with prior meta‐analysis

The findings of the present study build upon a previous meta‐analysis by Schluter et al. (2010), which also investigated the effects of exercise on cardiovascular outcomes in SHRs. Both studies found greater SBP reductions in younger SHRs compared to older animals and reported that exercise reduced RHR regardless of sex, age, or training duration (Schluter et al., 2010). Moreover, both analyses observed that female SHRs showed a blunted cardiovascular response to exercise compared to males. Several important differences, however, distinguish the two studies. Schlüter et al. included multiple exercise types including treadmill, free wheel running, and swimming, whereas the current meta‐analysis focused exclusively on treadmill‐based training. Our analysis also included a much larger dataset (116 vs. 18 studies). Schlüter et al. reported larger SBP reductions with shorter training durations, while our results demonstrated that longer exercise interventions yield greater improvements across all cardiovascular outcomes, including SBP. This discrepancy may stem from differences in study composition. For example, many of the longer‐duration studies in the Schlüter analysis were conducted in older SHRs, and mostly female SHRs, both of which, according to our analysis, exhibit blunted cardiovascular responses to exercise. Such physiological factors may have contributed to the reduced effectiveness of longer‐duration interventions in their analysis. Despite these differences, both studies reinforce the cardiovascular benefits of exercise in SHRs and highlight the need for further research exploring how sex, age, and exercise duration impact the therapeutic potential of exercise in SHRs.

### 4.2 Influence of sex

Hypertension exhibits well‐documented sex‐based differences. Men typically have a higher prevalence of hypertension than age‐matched premenopausal women, but the incidence in women rises substantially after menopause, eventually exceeding that of similarly aged men (Connelly et al., 2022; Virani et al., 2021). Moreover, hypertensive women often experience worse clinical outcomes, including higher rates of myocardial infarction and stroke (Connelly et al., 2022). Consistent with human data, the SHR model also displays pronounced sex‐specific differences in hypertension (Elmarakby & Sullivan, 2021). Fortepiani et al. reported that male SHRs have higher blood pressure than females until approximately 10–12 months of age, after which differences diminish, likely due to postmenopausal changes in females (Fortepiani et al., 2003). Our meta‐analysis supports these findings, showing that female SHRs began exercise training with significantly lower SBP compared to males (184.6 ± 4.7 mmHg vs. 200.6 ± 19.0 mmHg; *p* < 0.001). While males experienced a significant SBP reduction following exercise (to 180.4 ± 26.3 mmHg), females showed no significant change (182.6 ± 6.6 mmHg), resulting in comparable post‐training SBP between sexes. One explanation may be that female SHRs are closer to a physiological lower limit for SBP, below which further reductions are unlikely even with exercise, although further studies are needed to directly test this.

These results raise important questions about sex‐based exercise responsiveness. In humans, meta‐analyses have reported mixed results. Cornelissen and Smart (2013) found that men experienced more than twice the SBP reduction following dynamic endurance training, while Lu et al. (2021) reported greater benefits of high‐intensity interval training in men. In contrast, other studies observed no sex differences in response to either leisure‐time or total physical activity (Liu et al., 2017) or dynamic resistance exercise (Cornelissen & Smart, 2013). Thus, the clinical evidence remains inconclusive, and further research is needed to clarify sex‐specific responses. Moreover, whether certain exercise modalities are more effective in female SHRs remains unknown, an area our laboratory is actively investigating.

Physiological adaptations to exercise may also differ by sex. Trained women often exhibit distinct electrophysiological profiles (D'Ascenzi et al., 2020; La Gerche et al., 2022), different patterns of cardiac remodeling (Finocchiaro et al., 2017), and lower blood pressures during peak exercise effort (D'Ascenzi et al., 2020). In preclinical research, Coimbra et al. (2008) were among the first to report sex‐specific differences in SHR exercise responses. Their earlier work reported that male SHRs exhibited beneficial skeletal muscle arteriole remodeling after exercise, including reduced wall‐to‐lumen ratio, a marker of improved vascular function (Melo et al., 2003). In contrast, subsequent work demonstrated that female SHRs did not exhibit these vascular improvements and instead showed an increased wall‐to‐lumen ratio in renal arterioles (Coimbra et al., 2008). The authors suggested that these vascular remodeling differences may contribute to the persistence of elevated blood pressure in female SHRs despite training. Evidence also points to oxidative stress as a contributing factor, with sex‐specific roles in the development and maintenance of hypertension (Reckelhoff et al., 2019). Given our findings that females demonstrated smaller reductions in SBP and MAP compared with males, it is plausible that exercise has a diminished effect on oxidative stress mechanisms in females, thus contributing to the attenuated blood pressure response. Overall, these findings illustrate the complexity of sex‐specific exercise responses in this hypertensive model and underscore the need for targeted mechanistic research.

### 4.3 Impact of exercise intensity

Current clinical exercise guidelines for hypertension management are generalized rather than individualized, recommending 150 min of moderate‐intensity or 75 min of vigorous‐intensity exercise per week for all individuals, regardless of personal characteristics (Unger et al., 2020; Whelton et al., 2018). As a result, the optimal training regimen for people with hypertension remains an area of ongoing investigation, and exercise intensity is a particularly debated variable in humans (Igarashi & Nogami, 2020; Swain & Franklin, 2006) and SHRs. Our meta‐analysis indicates that low‐ and moderate‐intensity exercise produced greater cardiovascular benefits than high‐intensity protocols, with the largest effect seen in SBP reduction. Several mechanisms may explain the diminished benefits of high‐intensity exercise in SHRs, including exacerbated calcium channel dysfunction, limited improvements in oxidative stress, dysfunction of endothelial nitric oxide synthase (eNOS)‐nitric oxide (NO) signaling, and sustained sympathetic activation (Battault et al., 2016; Chen et al., 2019; Gava et al., 1995; Luo et al., 2021; Ye et al., 2019). Similar adverse outcomes have been noted with other exercise modalities. For example, da Costa Rebelo et al. (da Costa Rebelo et al., 2012) reported fibrotic cardiac remodeling after high‐intensity free‐wheel running. However, some high‐intensity treadmill studies in our analysis demonstrated beneficial adaptations, such as reduced renal inflammation and pro‐fibrotic signaling, suppression of cardiac apoptosis with enhanced pro‐survival signaling, protection against hypoperfusion/reperfusion contractile dysfunction, improved baroreflex sensitivity, and increased VO_2_ max and mitochondrial biogenesis (Brum et al., 2000; Crisman & Tomanek, 1985; Huang et al., 2012, 2018; Reger et al., 2006). Collectively, these mixed results reflect the complex and sometimes contradictory role of high‐intensity exercise in SHRs and emphasize the need for further research to clarify its mechanistic effects and translational relevance in hypertension.

### 4.4 Influence of age

The prevalence of hypertension increases sharply with advancing age in humans (Martin et al., 2024), and clinical studies demonstrate that exercise can meaningfully lower blood pressure among older adults with hypertension (Cornelissen & Smart, 2013; Igarashi et al., 2021; Kazeminia et al., 2020). Aging is associated with numerous cardiovascular changes that likely contribute to the development and progression of hypertension (Sun, 2015), yet only three studies in our dataset examined SHRs older than 8 months (Pagan et al., 2021; Quiroga et al., 2020; Rossoni et al., 2011). Rossoni et al. (Rossoni et al., 2011) observed that exercise reduced SBP and RHR in aged SHRs and improved myocardial capillary density, attenuated left ventricular hypertrophy, and reduced cardiac fibrosis. The other two studies found no SBP reduction but observed other cardiovascular benefits. For example, Pagan et al. (2021) reported improved papillary muscle contractility and increased antioxidant enzyme activity, while Quiroga et al. (2020) observed improvements in diastolic function. Collectively, these findings suggest that exercise benefits the aging SHR heart and vasculature even without lowering SBP. However, the small number of studies and variability in measured outcomes underscore the need for more research to define how age influences exercise responses in hypertension, ideally assessing a broader range of cardiovascular endpoints than those included in this meta‐analysis.

### 4.5 Role of training duration

Another key finding of this meta‐analysis is that shorter training durations were less effective in reducing MAP and yielded smaller reductions in both SBP and RHR in SHRs. This observation aligns with clinical results from Lee and Chae (2020), who reported greater blood pressure reductions with exercise programs longer than 8 weeks compared to shorter interventions (Lee & Chae, 2020). Based on our subgroup analysis, we recommend that exercise interventions in SHRs should be at least 8 weeks in duration to achieve meaningful cardiovascular benefits, with optimal effects observed at 8–12 weeks. In contrast, Cornelissen and Smart (2013) found that durations exceeding 24 weeks did not reduce SBP or diastolic blood pressure (DBP) in humans, although they attributed this to reduced supervision and participant adherence. Cao et al. (2020) concluded that more well‐designed studies are required to determine the optimal length of exercise interventions (Cao et al., 2020). No previous study has systematically compared training durations in SHRs; thus, making this the first meta‐analysis to evaluate their relationship in this model of hypertension. Further research should elucidate how training duration modulates the anti‐hypertensive effects of exercise, both in animal models and human populations.

### 4.6 Mechanisms impacted by exercise

A major strength of the SHR model is its utility in exploring the mechanisms underlying hypertension, making it a valuable tool for identifying therapeutic targets. Among the 116 studies included in this meta‐analysis, many examined mechanistic pathways alongside cardiovascular outcomes. Accumulating evidence from our included studies suggests that exercise lowers blood pressure through a range of mechanisms that exist in both SHRs and humans. Several included studies indicate that exercise improves vascular endothelial function and reduces vascular resistance by enhancing endothelial nitric oxide (NO) signaling and reducing oxidative stress (Cao et al., 2020; Gomes et al., 2019; Graham & Rush, 2004; Gu et al., 2013; Gu et al., 2014; Ito et al., 2013; Ye et al., 2019), leading to greater endothelial NO synthase activity, reduced reactive oxygen species (ROS), and upregulation of antioxidant enzymes such as superoxide dismutase and catalase (Bertagnolli et al., 2006; Blanco‐Rivero et al., 2013; Gomes et al., 2019; Mi et al., 2019; Moraes‐Teixeira Jde et al., 2010; Ren et al., 2016; Silva et al., 2017; Ye et al., 2019). Studies in our analysis also demonstrate that exercise improves autonomic regulation by reducing sympathetic nervous system activity, lowering sympathetic outflow (Bertagnolli et al., 2006; Brum et al., 2000; Chaar et al., 2015; Guo et al., 2008; Jia et al., 2014; Ren et al., 2016), and enhancing baroreflex function (Brum et al., 2000; Gu et al., 2013; Raimundo Fernades et al., 2020). Additionally, included studies reported exercise exerts anti‐inflammatory effects including decreases in systemic and tissue‐specific inflammation (Agarwal et al., 2009, 2011; Jia et al., 2014; Luo et al., 2021; Masson et al., 2014; Masson et al., 2015). Structural vascular adaptations were also observed in several studies, such as increased capillary density (Amaral et al., 2008; Coimbra et al., 2008; Guo et al., 2008) and reduced arterial wall thickness (Luo et al., 2021; Miotto et al., 2021; Peng et al., 2019; Rossoni et al., 2011; Ye et al., 2019; Zhang et al., 2019). At the cardiac level, studies showed that exercise attenuates left ventricular hypertrophy and decreases myocardial fibrosis (Lee et al., 2006; Miotto et al., 2021; Peng et al., 2019; Rossoni et al., 2011). Finally, multiple included studies found that exercise modulates the renin‐angiotensin system, both in the central nervous system and peripheral tissues, restoring vascular homeostasis by promoting vasodilation and reducing hypertensive signaling (Chaar et al., 2015; da Costa et al., 2020; Felix & Michelini, 2007; Gu et al., 2014; Peng et al., 2019; Ren et al., 2016). Collectively, these findings from our included studies illustrate the multifaceted, synergistic mechanisms through which exercise likely exerts cardiovascular benefits in SHRs, supporting its role in both the prevention and management of the disease.

### 4.7 Exercise non‐responders

The variability in exercise responsiveness observed in our analysis, including the absence of cardiovascular benefits in some studies, particularly in certain subgroups, highlights the need for strategies to address exercise non‐responsiveness. Human studies suggest that switching exercise modalities can benefit individuals who do not respond to an initial training approach. For example, Ferreira et al. (2024) found that hypertensive postmenopausal women who failed to reduce blood pressure with aerobic training experienced significant improvements after transitioning to a different form of exercise. To our knowledge, this concept has not been investigated in SHRs, especially in females, and represents an important and underexplored direction for future research. Investigating such training sequences in SHRs could reveal mechanisms of non‐responsiveness and may inform more personalized and effective exercise interventions for the treatment of hypertension.

### 4.8 Limitations

Several limitations should be considered when interpreting these findings. Some subgroup analyses were based on small sample sizes, warranting cautious interpretation across all subgroups. For example, only 11 of the 116 studies involved female SHRs, and none directly compared male and female responses. Similarly, few studies examined aged SHRs or training durations shorter than 8 weeks or longer than 20 weeks. This limited evidence restricts our ability to determine how sex, age, or training length influence exercise responsiveness, highlighting the need for targeted research in each area. Notably, none of the female‐inclusive studies were published after the NIH policy requiring the inclusion of both sexes in preclinical research (Clayton & Collins, 2014).

Second, funnel plot analyses suggest the presence of publication bias or missing studies. However, even with imputed data using the trim‐and‐fill method, exercise training maintained a moderate effect across all three cardiovascular outcomes. Third, this meta‐analysis focused exclusively on treadmill‐based exercise training, limiting generalizability to other exercise modalities. To address this limitation, our laboratory is currently conducting a separate meta‐analysis examining cardiovascular responses to a broader range of exercise interventions in SHRs. Fourth, variability in the methodological factors, including exercise training progression (e.g., time to reach maximum speed or VO_2_max), environmental conditions (e.g., temperature, housing), and timing of outcomes measurements, may have introduced heterogeneity that was not fully accounted for in subgroup analyses. Additionally, risk of bias assessment revealed that many studies received “unclear” ratings across multiple domains, suggesting insufficient methodological detail in published reports rather than definitively poor study quality. This limits our ability to confidently assess whether observed effects genuinely reflect exercise training benefits or methodological issues. Despite these limitations, sensitivity analyses confirmed that no single study disproportionately affected the pooled effect estimates, supporting the robustness of our overall findings.

Finally, while SHRs are a widely used model of hypertension, the translational relevance of our findings remains uncertain. Although the SHR model exhibits many key features of human hypertension, it does not fully capture the complex pathophysiological and clinical heterogeneity seen in humans (Lerman et al., 2019), warranting caution in generalizing these results. Our analyses suggest that low‐ and moderate‐intensity exercise yields greater benefits in SHRs, whereas current clinical guidelines emphasize moderate‐ to high‐intensity exercise for blood pressure reduction (Unger et al., 2020; Whelton et al., 2002). The basis for this discrepancy remains unclear and may reflect fundamental physiological differences or methodological variations in exercise implementation. For example, most studies in SHRs include a younger population, whereas clinical studies typically include older hypertensive humans. Future studies directly comparing intensity‐response relationships in SHRs and humans could help resolve this gap and improve the translational relevance of preclinical findings. It is also unclear whether the sex‐ and age‐based differences observed in SHRs translate directly to hypertensive humans undergoing similar training protocols. Nevertheless, our findings emphasize the importance of considering biological variables such as sex and age in both preclinical and clinical exercise research for hypertension.

## 5 CONCLUSION

In summary, this meta‐analysis is the first to quantitatively evaluate the effects of treadmill‐based exercise training on key cardiovascular outcomes including SBP, MAP, and RHR in SHRs. These results provide robust evidence that exercise training significantly improves all three parameters, reinforcing its important role as a nonpharmacological intervention in the prevention and management of hypertension. Subgroup analyses further reveal that SBP reductions are moderated by exercise intensity, sex, and age; MAP improvements are influenced by sex, exercise intensity, and training duration; and RHR decreases are primarily affected by exercise intensity. Notably, the limited number of studies involving female and aged SHRs represents a critical gap in the preclinical literature and underscores the need for future investigations to clarify how sex and age modulate the cardiovascular response to exercise training in this model. In summary, these findings have important implications for both preclinical research design and the translation of exercise‐based interventions for hypertension management.

## AUTHOR CONTRIBUTIONS

Conceived and designed research: Stephen W. Luckey and Kayla M. Meredith. Performed experiments: Stephen W. Luckey, Kayla M. Meredith, Natalie S. Crouse, and Rachael Bush. Analyzed data: Stephen W. Luckey, Natalie S. Crouse, and RS. Interpreted results of experiments: Stephen W. Luckey, Natalie S. Crouse, Rohan Sethi, and Rachael Bush. Prepared figures: Stephen W. Luckey and Rohan Sethi. Drafted manuscript: Stephen W. Luckey. Edited and revised manuscript: Stephen W. Luckey, Kayla M. Meredith, Natalie S. Crouse, and Rohan Sethi. Approved final version of the manuscript: Stephen W. Luckey, Kayla M. Meredith, Natalie S. Crouse, Rohan Sethi, and Rachael Bush.

## FUNDING INFORMATION

The authors have nothing to report.

## CONFLICT OF INTEREST STATEMENT

The authors declare no conflicts of interest.

## ETHICS STATEMENT

All data were obtained from peer‐reviewed, published original studies; therefore, no additional ethical approval was required.

## Supporting information


**Figure S1.** PRISMA checklist.

**Figure S2.** Summary of risk of bias assessment using the SYRCLE tool across all included studies. Each bar represents the percentage of studies rated as low risk (green), unclear risk (yellow), or high risk (red) for each of the 10 SYRCLE domains.

**Figure S3.** Funnel plot of systolic blood pressure (SBP) outcomes in exercised spontaneously hypertensive rates (SHRs). Open circles represent observed studies; black circles represent studies imputed using trim‐and‐fill method.

**Figure S4.** Forest plot depicting the effect of training duration on systolic blood pressure (SBP) in spontaneously hypertensive rates (SHRs), with subgroup analysis evaluating the impact of ≤8 weeks, 9–12 weeks, and ≥13 weeks of training. Standardized mean differences (Hedges’ *g*) with 95% confidence intervals (CI)s are shown for individual studies. The overall pooled effect size, calculated using a random effects model, is represented by the diamond at the bottom. Values to the left of zero indicate a beneficial effect of exercise training (Favors EX), whereas values to the right indicate a greater response in the sedentary group (Favors SED). Studies with letter designations (L/M/H, low/moderate/high intensity; M/F, male/female) indicate publications with multiple experimental groups, each analyzed separately. *p*‐values displayed as ‘*p* = 0.000’ indicate *p* < 0.001.

**Figure S5.** Funnel plot of mean arterial pressure (MAP) outcomes in exercised spontaneously hypertensive rates (SHRs). Open circles represent observed studies; black circles represent studies imputed using trim‐and‐fill method.

**Figure S6.** Forest plot depicting the effect of age at the onset of exercise on mean arterial pressure (MAP) in spontaneously hypertensive rates (SHRs), with subgroup analysis evaluating the impact of age at the prehypertensive, developing hypertension, and hypertensive stages. Standardized mean differences (Hedges’ *g*) with 95% confidence intervals (CI)s are shown for individual studies. The overall pooled effect size, calculated using a random effects model, is represented by the diamond at the bottom. Values to the left of zero indicate a beneficial effect of exercise training (Favors EX), whereas values to the right indicate a greater response in the sedentary group (Favors SED). Studies with letter designations (L/M/H, low/moderate/high intensity; M/F, male/female; C/T, catheter/tail cuff) indicate publications with multiple experimental groups, each analyzed separately. *p*‐values displayed as ‘*p* = 0.000’ indicate *p* < 0.001.

**Figure S7.** Funnel plot of resting heart rate (RHR) outcomes in exercised spontaneously hypertensive rates (SHRs). Open circles represent observed studies; black circles represent studies imputed using trim‐and‐fill method.

**Figure S8.** Forest plot depicting the effect of sex on resting heart rate (RHR) in spontaneously hypertensive rates (SHRs), with subgroup analysis evaluating differences between males and females. Standardized mean differences (Hedges’ *g*) with 95% confidence intervals (CI)s are shown for individual studies. The overall pooled effect size, calculated using a random effects model, is represented by the diamond at the bottom. Values to the left of zero indicate a beneficial effect of exercise training (Favors EX), whereas values to the right indicate a greater response in the sedentary group (Favors SED). Studies with letter designations (L/M/H, low/moderate/high intensity; M/F, male/female; C/T, catheter/tail cuff) indicate publications with multiple experimental groups, each analyzed separately. *p*‐values displayed as ‘*p* = 0.000’ indicate *p* < 0.001.

**Figure S9.** Forest plot depicting the effect of age at the onset of exercise on resting heart rate (RHR) in spontaneously hypertensive rates (SHRs), with subgroup analysis evaluating the impact of age at the prehypertensive, developing hypertension, and hypertensive stages. Standardized mean differences (Hedges’ *g*) with 95% confidence intervals (CI)s are shown for individual studies. The overall pooled effect size, calculated using a random effects model, is represented by the diamond at the bottom. Values to the left of zero indicate a beneficial effect of exercise training (Favors EX), whereas values to the right indicate a greater response in the sedentary group (Favors SED). Studies with letter designations (L/M/H, low/moderate/high intensity; M/F, male/female; C/T, catheter/tail cuff) indicate publications with multiple experimental groups, each analyzed separately. *p*‐values displayed as ‘*p* = 0.000’ indicate *p* < 0.001.

**Figure S10.** Forest plot depicting the effect of training duration on resting heart rate (RHR) in spontaneously hypertensive rates (SHRs), with subgroup analysis evaluating the impact of ≤8 weeks, 9–12 weeks, and ≥13 weeks of training. Standardized mean differences (Hedges’ *g*) with 95% confidence intervals (CI)s are shown for individual studies. The overall pooled effect size, calculated using a random effects model, is represented by the diamond at the bottom. Values to the left of zero indicate a beneficial effect of exercise training (Favors EX), whereas values to the right indicate a greater response in the sedentary group (Favors SED). Studies with letter designations (L/M/H, low/moderate/high intensity; M/F, male/female; C/T, catheter/tail cuff) indicate publications with multiple experimental groups, each analyzed separately. *p*‐values displayed as ‘*p* = 0.000’ indicate *p* < 0.001.

## Data Availability

Data will be available upon reasonable request.
