# Supplementary figures and images for: Cardiovascular effects of exercise training in spontaneously hypertensive rats: A systematic review and meta‐analysis
Source: Physiol Rep. 2026 Mar 3;14(5):e70794. doi: 10.14814/phy2.70794 (PMC12956849; doi:10.14814/phy2.70794)

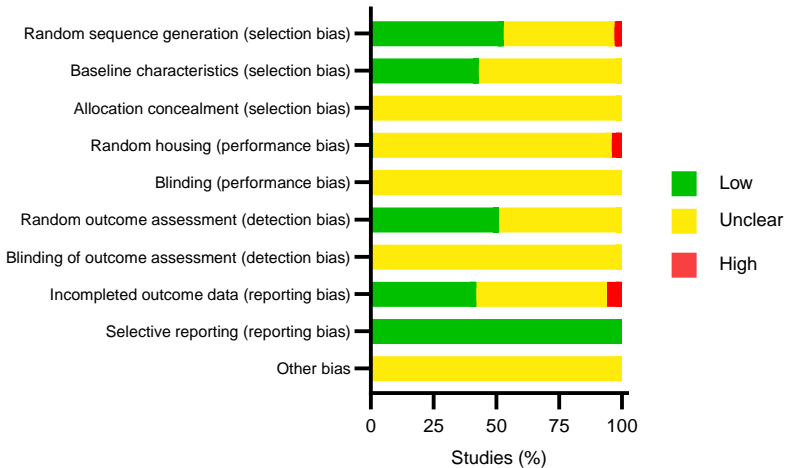

Supplement: Supplementary file 2 — Figure S2. Summary of risk of bias assessment using the SYRCLE tool across all included studies. Each bar represents the percentage of studies rated as low risk (green), unclear risk (yellow), or high risk (red) for each of the 10 SYRCLE domains. [file PHY2-14-e70794-s003.pdf]

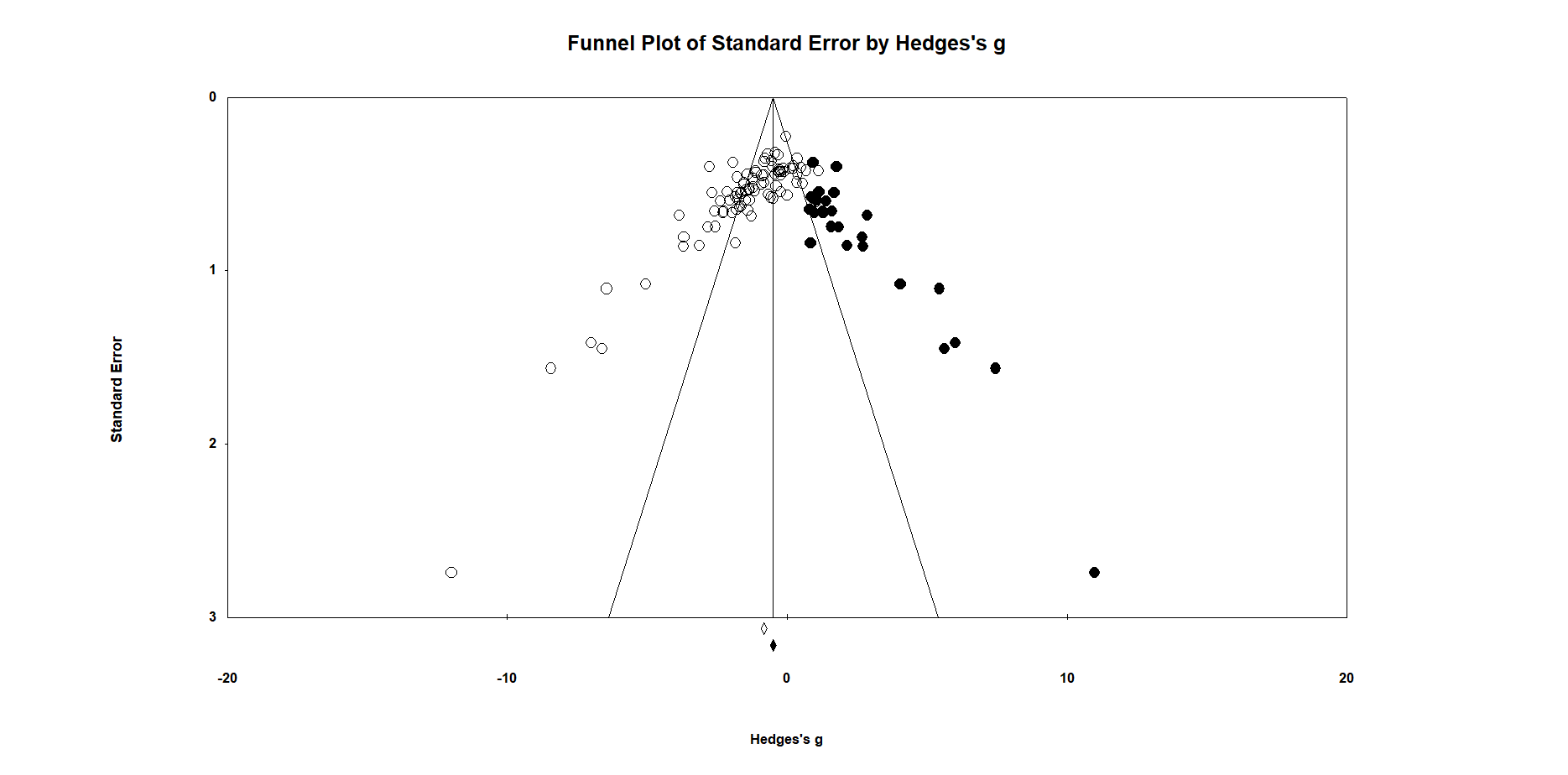

Supplement: Supplementary file 3 — Figure S3. Funnel plot of systolic blood pressure (SBP) outcomes in exercised spontaneously hypertensive rates (SHRs). Open circles represent observed studies; black circles represent studies imputed using trim‐and‐fill method. [file PHY2-14-e70794-s004.tif]

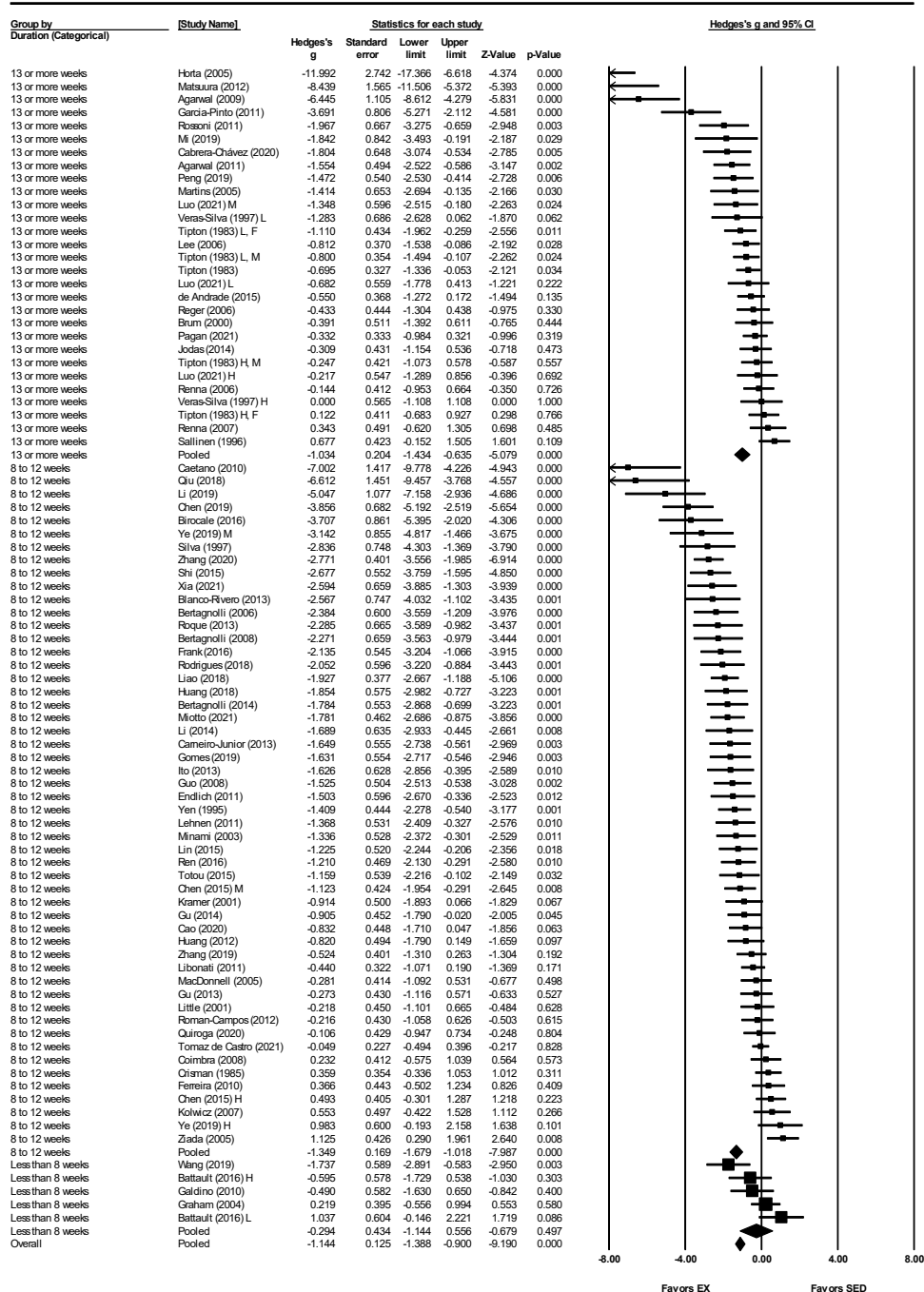

Supplement: Supplementary file 4 — Figure S4. Forest plot depicting the effect of training duration on systolic blood pressure (SBP) in spontaneously hypertensive rates (SHRs), with subgroup analysis evaluating the impact of ≤8 weeks, 9–12 weeks, and ≥13 weeks of training. Standardized mean differences (Hedges’ g) with 95% confidence intervals (CI)s are shown for individual studies. The overall pooled effect size, calculated using a random effects model, is represented by the diamond at the bottom. Values to the left of zero indicate a beneficial effect of exercise training (Favors EX), whereas values to the right indicate a greater response in the sedentary group (Favors SED). Studies with letter designations (L/M/H, low/moderate/high intensity; M/F, male/female) indicate publications with multiple experimental groups, each analyzed separately. p‐values displayed as ‘p = 0.000’ indicate p < 0.001. [file PHY2-14-e70794-s008.pdf]

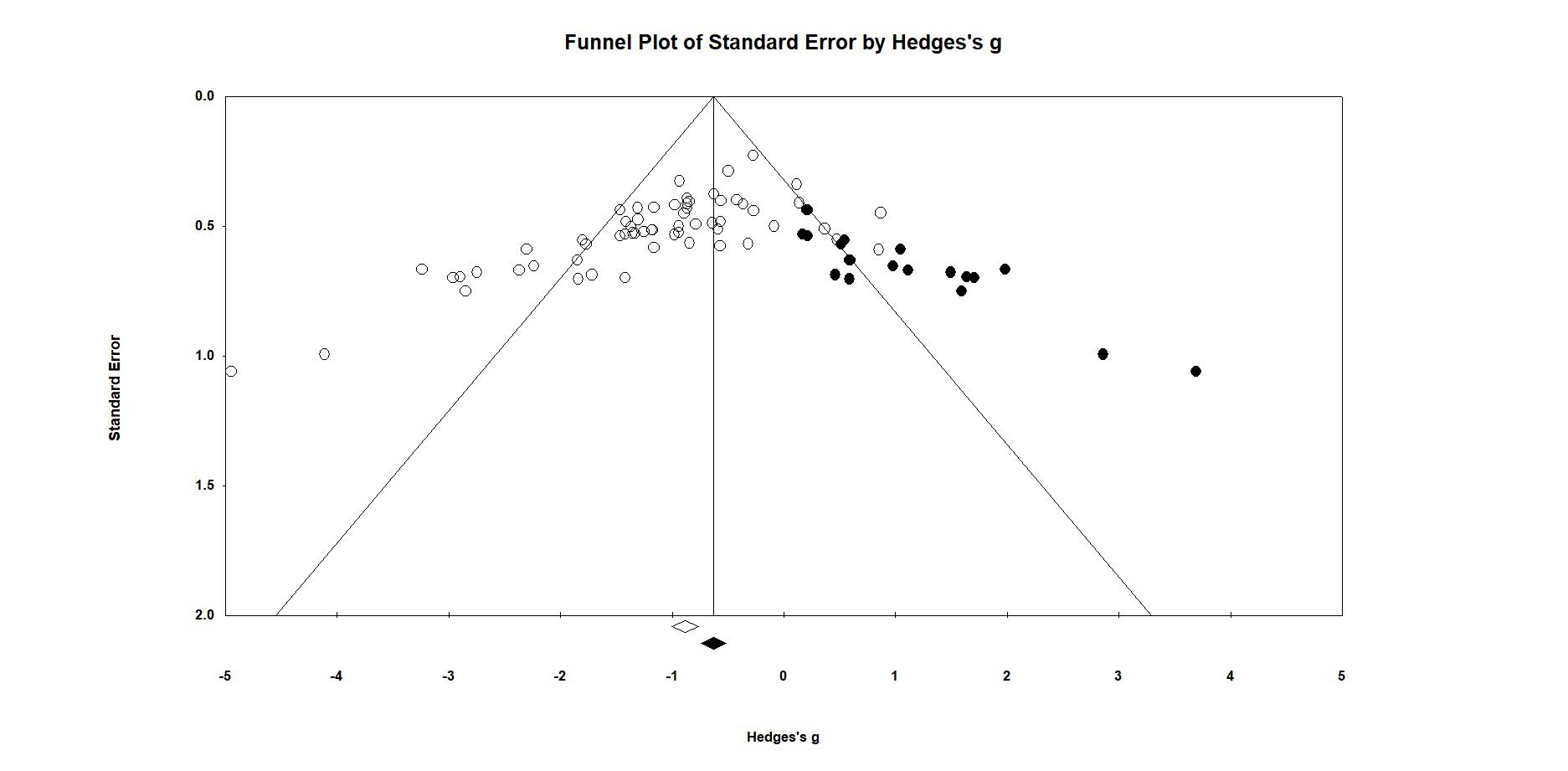

Supplement: Supplementary file 5 — Figure S5. Funnel plot of mean arterial pressure (MAP) outcomes in exercised spontaneously hypertensive rates (SHRs). Open circles represent observed studies; black circles represent studies imputed using trim‐and‐fill method. [file PHY2-14-e70794-s005.tif]

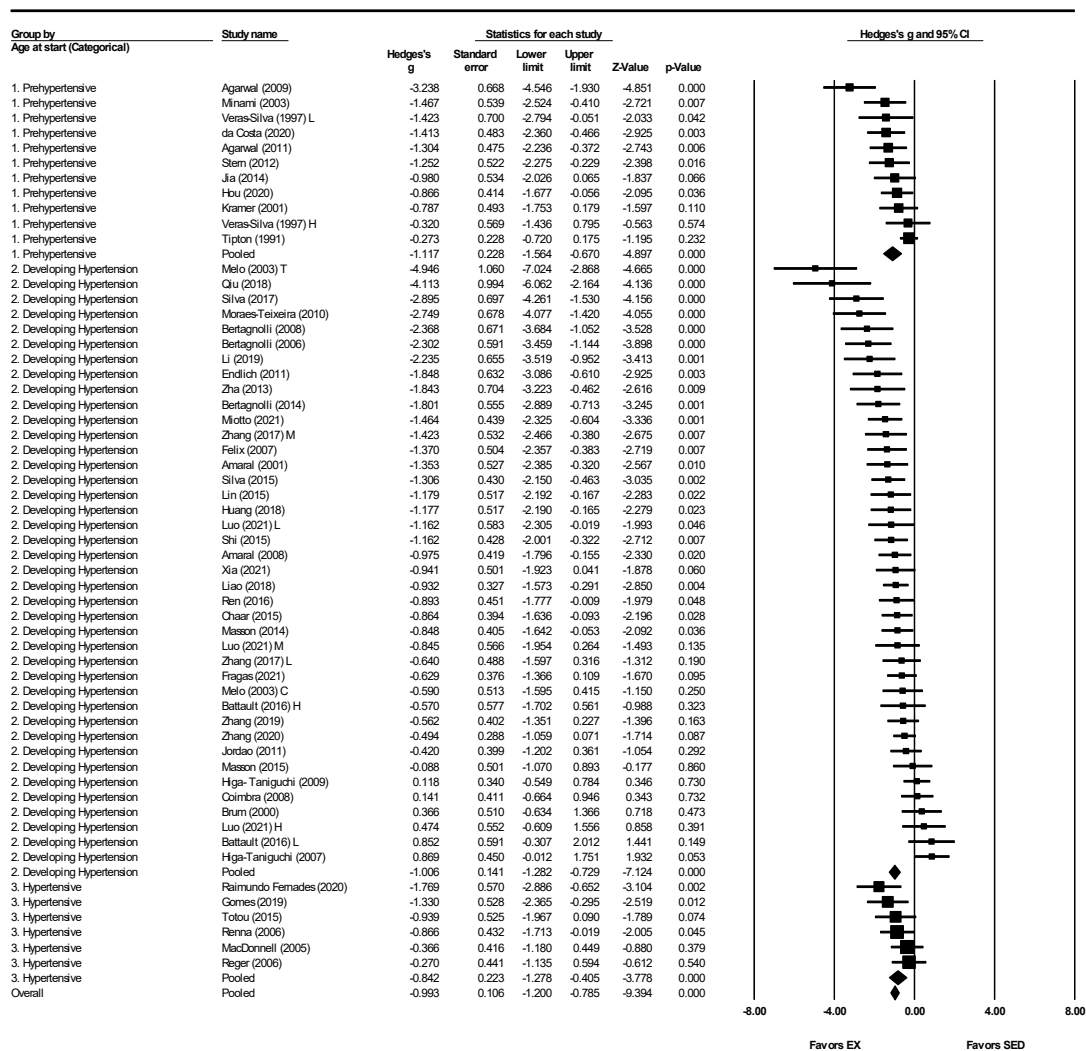

Supplement: Supplementary file 6 — Figure S6. Forest plot depicting the effect of age at the onset of exercise on mean arterial pressure (MAP) in spontaneously hypertensive rates (SHRs), with subgroup analysis evaluating the impact of age at the prehypertensive, developing hypertension, and hypertensive stages. Standardized mean differences (Hedges’ g) with 95% confidence intervals (CI)s are shown for individual studies. The overall pooled effect size, calculated using a random effects model, is represented by the diamond at the bottom. Values to the left of zero indicate a beneficial effect of exercise training (Favors EX), whereas values to the right indicate a greater response in the sedentary group (Favors SED). Studies with letter designations (L/M/H, low/moderate/high intensity; M/F, male/female; C/T, catheter/tail cuff) indicate publications with multiple experimental groups, each analyzed separately. p‐values displayed as ‘p = 0.000’ indicate p < 0.001. [file PHY2-14-e70794-s010.pdf]

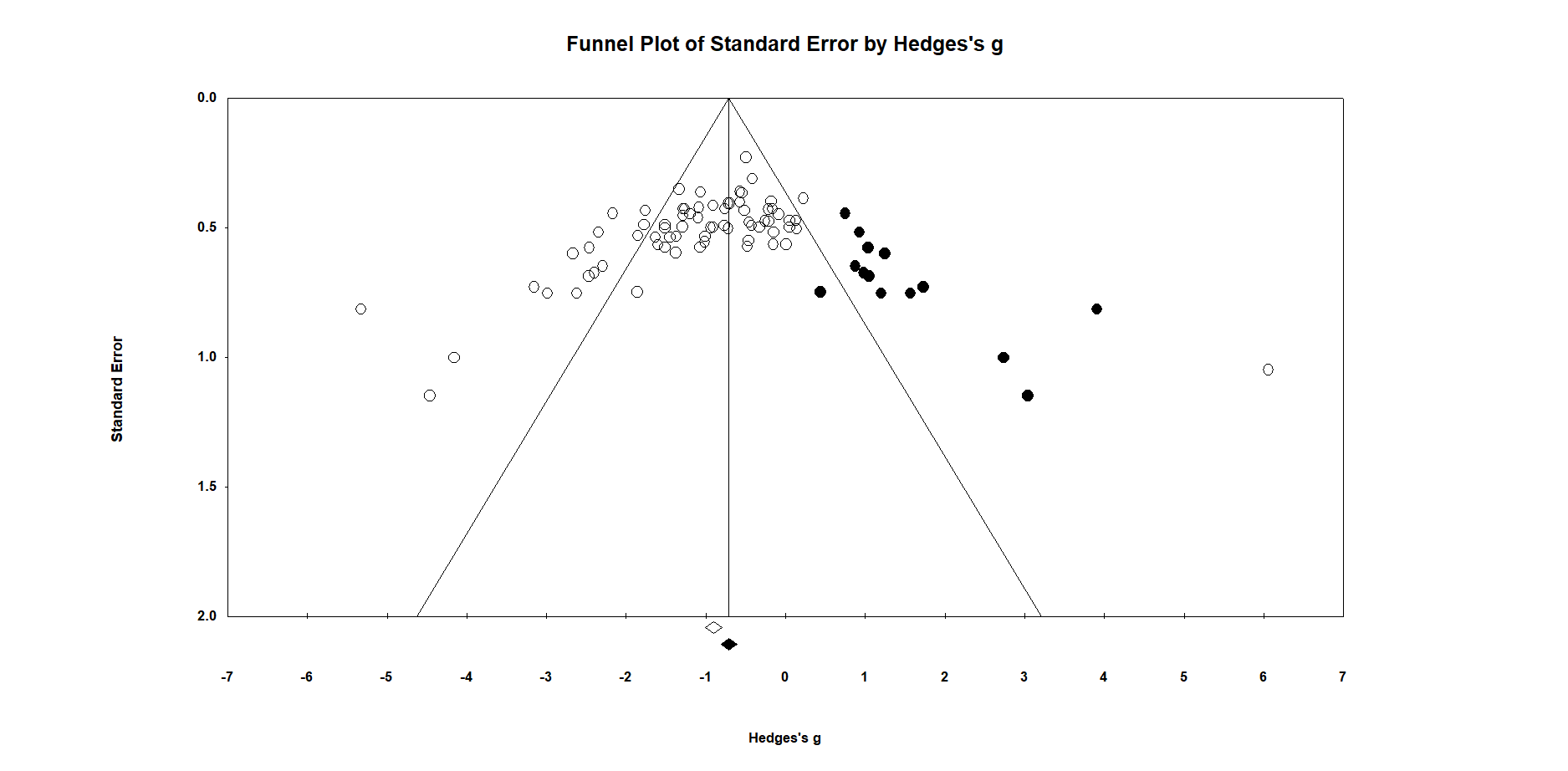

Supplement: Supplementary file 7 — Figure S7. Funnel plot of resting heart rate (RHR) outcomes in exercised spontaneously hypertensive rates (SHRs). Open circles represent observed studies; black circles represent studies imputed using trim‐and‐fill method. [file PHY2-14-e70794-s006.tif]

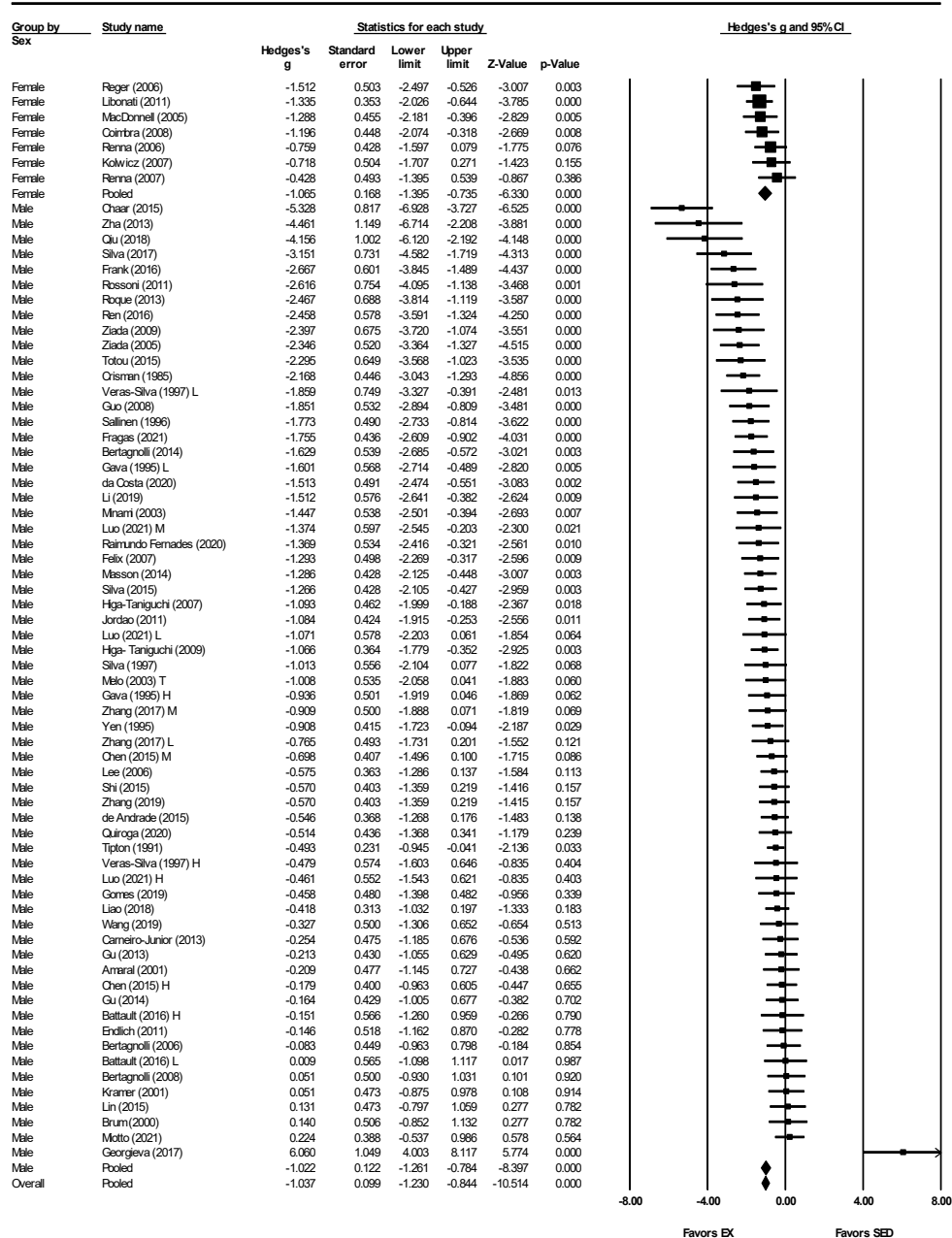

Supplement: Supplementary file 8 — Figure S8. Forest plot depicting the effect of sex on resting heart rate (RHR) in spontaneously hypertensive rates (SHRs), with subgroup analysis evaluating differences between males and females. Standardized mean differences (Hedges’ g) with 95% confidence intervals (CI)s are shown for individual studies. The overall pooled effect size, calculated using a random effects model, is represented by the diamond at the bottom. Values to the left of zero indicate a beneficial effect of exercise training (Favors EX), whereas values to the right indicate a greater response in the sedentary group (Favors SED). Studies with letter designations (L/M/H, low/moderate/high intensity; M/F, male/female; C/T, catheter/tail cuff) indicate publications with multiple experimental groups, each analyzed separately. p‐values displayed as ‘p = 0.000’ indicate p < 0.001. [file PHY2-14-e70794-s007.pdf]

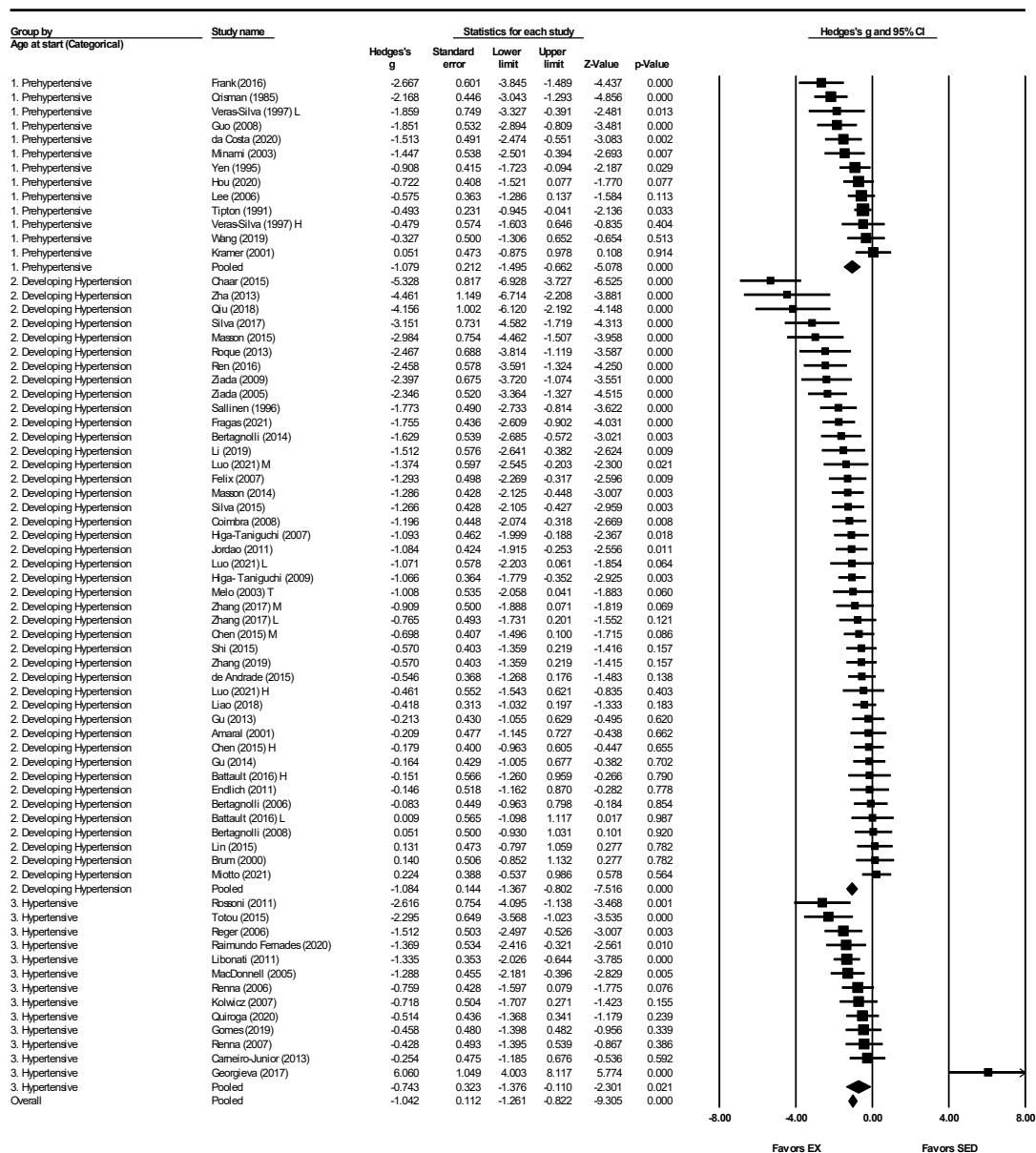

Supplement: Supplementary file 9 — Figure S9. Forest plot depicting the effect of age at the onset of exercise on resting heart rate (RHR) in spontaneously hypertensive rates (SHRs), with subgroup analysis evaluating the impact of age at the prehypertensive, developing hypertension, and hypertensive stages. Standardized mean differences (Hedges’ g) with 95% confidence intervals (CI)s are shown for individual studies. The overall pooled effect size, calculated using a random effects model, is represented by the diamond at the bottom. Values to the left of zero indicate a beneficial effect of exercise training (Favors EX), whereas values to the right indicate a greater response in the sedentary group (Favors SED). Studies with letter designations (L/M/H, low/moderate/high intensity; M/F, male/female; C/T, catheter/tail cuff) indicate publications with multiple experimental groups, each analyzed separately. p‐values displayed as ‘p = 0.000’ indicate p < 0.001. [file PHY2-14-e70794-s002.pdf]

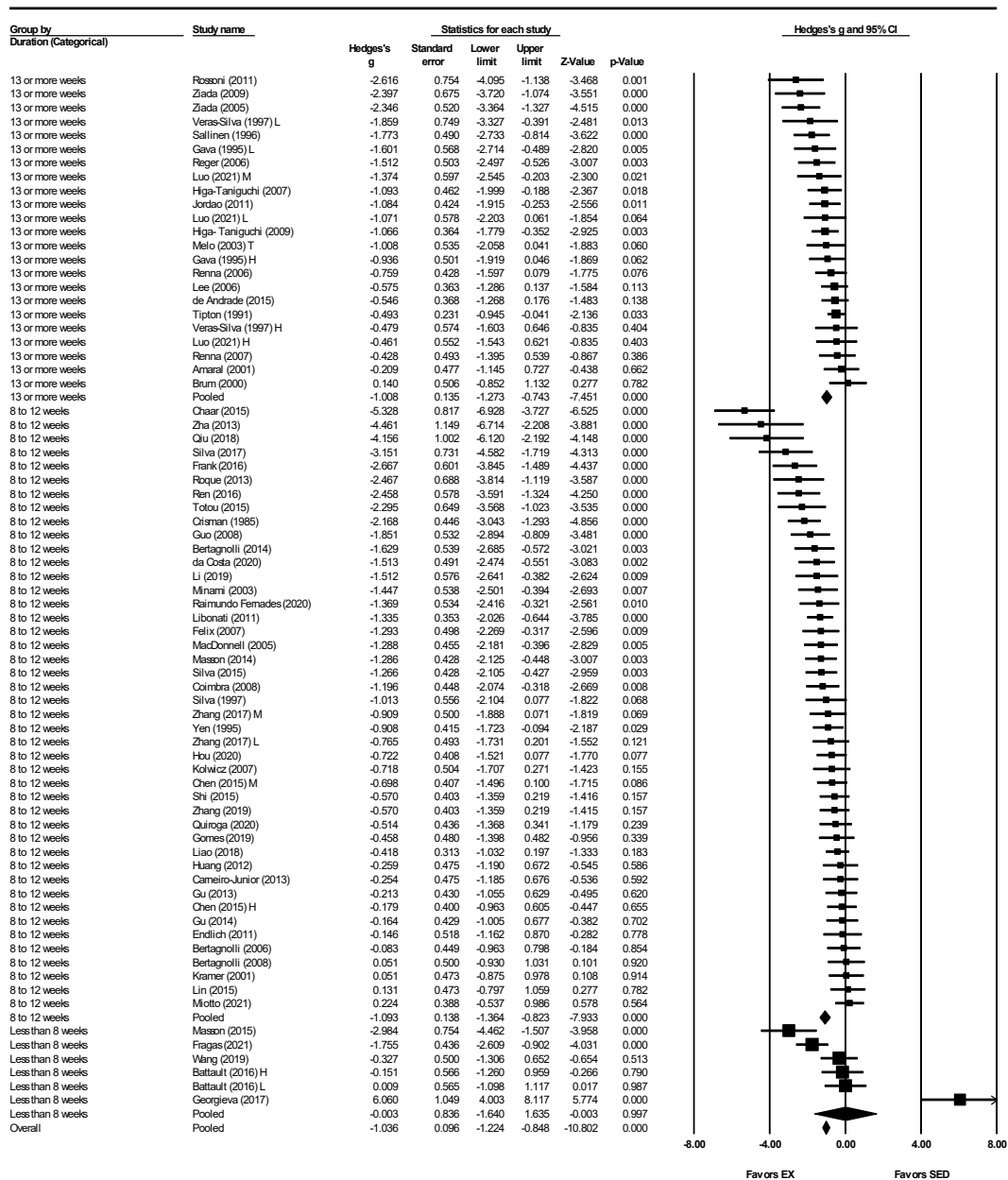

Supplement: Supplementary file 10 — Figure S10. Forest plot depicting the effect of training duration on resting heart rate (RHR) in spontaneously hypertensive rates (SHRs), with subgroup analysis evaluating the impact of ≤8 weeks, 9–12 weeks, and ≥13 weeks of training. Standardized mean differences (Hedges’ g) with 95% confidence intervals (CI)s are shown for individual studies. The overall pooled effect size, calculated using a random effects model, is represented by the diamond at the bottom. Values to the left of zero indicate a beneficial effect of exercise training (Favors EX), whereas values to the right indicate a greater response in the sedentary group (Favors SED). Studies with letter designations (L/M/H, low/moderate/high intensity; M/F, male/female; C/T, catheter/tail cuff) indicate publications with multiple experimental groups, each analyzed separately. p‐values displayed as ‘p = 0.000’ indicate p < 0.001. [file PHY2-14-e70794-s009.pdf]
